# Oestrogen suppresses the adipogenesis of fibro/adipogenic progenitors through reactivating the METTL3–ESR1‐mediated loop in post‐menopausal females

**DOI:** 10.1002/ctm2.70206

**Published:** 2025-01-28

**Authors:** Hao Zhou, Shujing Feng, Jinkui Cai, Xiexiang Shao, Siyuan Zhu, Han Zhou, Yongmin Cao, Ru Wang, Xingzuan Lin, Jianhua Wang

**Affiliations:** ^1^ Xinhua Hospital Affiliated to Shanghai Jiaotong University School of Medicine Shanghai China; ^2^ School of Exercise and Health Shanghai University of Sport Shanghai China; ^3^ Wuhan Third Hospital Tongren Hospital of Wuhan University Wuhan China; ^4^ Department of Hand Surgery Huashan Hospital Fudan University Shanghai China; ^5^ Peking University Third Hospital Beijing China

**Keywords:** adipogenesis, ESR1, fibro/adipogenic progenitors (FAPs), m6A methylation, METTL3, muscular fatty infiltration

## Abstract

**Background:**

Post‐menopausal women experience more severe muscular fatty infiltration, though the mechanisms remain unclear. The decline in estrogen levels is considered as a critical physiological alteration during post‐menopause. Fibro/adipogenic progenitors (FAPs) are identified as major contributors to muscular fatty infiltration. This study aimed to investigate the detailed mechanism underlying the excessive muscular fatty infiltration in postmenopausal females.

**Methods:**

Supraspinatus muscle samples were collected from female patients with or without menopause, and from mice with or without ovariectomy (OVX), to evaluate muscular fatty infiltration and isolated FAPs. The expressions of (estrogen receptor 1) ESR1, methyltransferase‐like 3 (METTL3), and adipogenesis ability in FAPs from post‐menopausal women and OVX mice were investigated. RNA sequencing (RNA‐Seq) was performed to explore the gene expression profiles and potential mechanisms in FAPs from Pdgfrα‐CreERT2; Esr1 knockout (Esr1 KO) mice and Esr1 flox/flox (Esr1 f/f) mice. The interplay of the METTL3‐ESR1 mediated loop and its role in regulating adipogenesis in FAPs were investigated using dual luciferase reporter assays, chromatin immunoprecipitation (ChIP), and protein and RNA stability assays. The effects of estrogen supplementation on muscular fatty infiltration and locomotor function in OVX mice were evaluated by immunofluorescent staining and functional analysis.

**Results:**

Decreased expression of ESR1/METTL3 and increased adipogenesis ability in FAPs was found in post‐menopausal female. METTL3‐mediated m6A methylation promoted ESR1 mRNA stability at the post‐transcriptional level in FAPs. METTL3‐mediated m6A modification promoted ESR1 expression by stabilizing ESR1 mRNA, while ESR1 acted as a transcription factor that enhanced METTL3 transcription in turn. ESR1 also suppressed the transcription of the adipogenic transcription factor peroxisome proliferator‐activated receptor gamma (PPARγ), thereby inhibiting adipogenesis in FAPs. Reactivation of the METTL3‐ESR1 mediated loop by estrogen alleviated excessive adipogenesis in FAPs from post‐menopausal women, and it also reduced muscular fatty infiltration, and improved locomotor function in OVX mice.

**Conclusion:**

Excessive muscular fatty infiltration in post‐menopausal women arose from the disruption of the METTL3‐ESR1 mediated loop of FAPs due to estrogen deficiency. Reactivation of the METTL3‐ESR1 mediated loop by estrogen may serve as a novel intervention to inhibit excessive adipogenesis of post‐menopausal female FAPs, thereby ameliorating muscular fatty infiltration and improving locomotor function in post‐menopausal females.

**Key points:**

- Oestrogen insufficiency disrupted the METTL3ESR1 loop in post‐menopausal FAPs, causing excessive muscular fatty infiltration.
- METTL3‐mediated m6A modification stabilized ESR1 mRNA and enhanced ESR1 expression, while increased ESR1 further promoted METTL3 transcription.
- ESR1 inhibited the transcription of adipogenic factor PPARγ, ameliorating adipogenesis in FAPs.
- Reactivating the METTL3ESR1 loop via oestrogen in FAPs reduced muscular fatty infiltration and improved locomotor function.

## Graphical abstract

Excessive muscular fatty infiltration in post‐menopausal women arose from the disruption of the methyltransferase‐like 3 (METTL3)–oestrogen receptor 1 (ESR1)‐mediated loop of fibro/adipogenic progenitors (FAPs) due to oestrogen deficiency. Reactivation of the METTL3–ESR1‐mediated loop by oestrogen alleviated excessive adipogenesis in FAPs, thereby ameliorating muscular fatty infiltration and improving locomotor function in ovariectomy (OVX) mice.

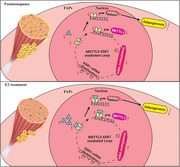

## 1 INTRODUCTION

Muscular fatty infiltration refers to ectopic fat deposits within muscle fibres and around muscle bundles.^1^ Low levels of muscular fatty infiltration are considered a normal component of adult muscle without pathological effects.^1^ However, elevated muscular fatty infiltration is frequently linked to musculoskeletal imbalance and impaired locomotion, increasing the risk of falls and worsening functional outcomes.^2^, ^3^, ^4^, ^5^, ^6^, ^7^ Importantly, the likelihood and severity of fatty infiltration escalate with age,^8^ particularly among post‐menopausal women.^3^, ^9^, ^10^ However, the detailed mechanisms underlying excessive muscular fatty infiltration in post‐menopausal women remain unclear, with a dearth of specific treatment strategies targeting muscular fatty infiltration.

Fibro/adipogenic progenitors (FAPs) are a cluster of stem cells expressing specific marker PDGFRα in skeletal muscles, playing pivotal roles in regulating muscle fatty degeneration, fibrosis, regeneration and maintaining microenvironment stability.^1^, ^11^, ^12^, ^13^ Previous studies have demonstrated that muscular fatty infiltration is derived from FAPs.^1^, ^14^ During acute injury, FAPs are efficiently eliminated through the strict regulation of various immune cells.^15^ However, in chronic muscle diseases, FAPs primarily contribute to muscular fatty infiltration and fibrosis, including Duchenne muscular dystrophy (DMD), prolonged denervation, inflammatory myopathies and trauma.^1^, ^16^, ^17^ Significant advancement in preventing muscular fatty infiltration has been achieved by modulating localised signalling cascades, epigenetic modifications, complex interactions with immune cells and inflammatory mediators for FAPs.^9^, ^12^, ^13^, ^18^ Hence, FAPs serve as crucial target cells for treating muscular fatty infiltration. However, the specific mechanism of FAPs in contributing increased muscular fatty infiltration in post‐menopausal women also remains elusive.

Post‐menopausal women suffered from marked epigenetic modifications changes^19^, ^20^ and reduced oestrogen levels.^21^ Previous studies have demonstrated that skeletal muscle atrophy, fatty accumulation and impaired metabolic homeostasis were associated with epigenetic changes in post‐menopausal women.^22^, ^23^, ^24^, ^25^ Oestrogen regulates biological effects by binding to oestrogen receptor (ESR),^26^ and numerous studies also suggested that the oestrogen receptor 1 (ESR1) was down‐regulated in post‐menopause, which significantly contributed to muscle pathophysiological alterations.^27^, ^28^, ^29^, ^30^, ^31^ Therefore, oestrogen‐related epigenetic modifications may play a critical role in muscular fatty infiltration following menopause.

This study aimed to elucidate the mechanisms underlying the excessive muscular fatty infiltration in post‐menopausal females. Decreased expression of ESR1/methyltransferase‐like 3 (METTL3) and increased adipogenesis ability in FAPs was found in post‐menopausal female. METTL3‐mediated N6‐methyladenosine (m6A) modification promoted ESR1 expression by stabilising ESR1 mRNA, while ESR1 acted as a transcription factor that enhanced METTL3 transcription in turn. ESR1 also suppressed the transcription of the adipogenic transcription factor peroxisome proliferator‐activated receptor gamma (PPARγ), thereby inhibiting adipogenesis in FAPs. Reactivation of the METTL3–ESR1‐mediated loop by oestrogen alleviated excessive adipogenesis in FAPs from post‐menopausal women, and it also reduced muscular fatty infiltration, and improved locomotor function in ovariectomy (OVX) mice.

## 2 MATERIALS AND METHODS

### 2.1 Human sample

The local Ethics Committee granted approval for this study (Approval Nos. XHEC‐D‐2022‐129 and XHEC‐D‐2024‐073‐1). Human supraspinatus muscle samples were collected from patients who underwent shoulder arthroplasty or experienced shoulder trauma between July 2022 and July 2024, as previously described.^32^ Post‐menopausal inclusion criteria required 12 consecutive months of amenorrhea without intervention.^33^ Patients were assigned to the peri‐menopausal group based on their age, hormone levels and menstrual cycle in accordance with the established guidelines (STRAW+ 10).^33^ All participants signed informed consents before gathering clinical specimens. The subject characteristics were shown in Table S1.

### 2.2 Animals

Animal experiments adhered to the local Institutional Animal Welfare Committee guidelines (Approval No. XHEC‐F‐2023‐029). Pdgfrα‐CreERT2 mice (Jackson Laboratory, Strain NO. 032770) and Esr1f/f mice (Gem Pharmatech, Strain NO. T052334) were used. Gene knockout (KO) in FAPs was induced via intraperitoneal (i.p.) tamoxifen (TAM) injection (ABCONE, cat#T56488) every 48 h for 1 week.

To model menopause, OVX was performed on 8‐week‐old female C57BL/6J mice, following established protocols.^34^ Briefly, the ovaries were exposed by a double dorsolateral incision at the area between the last rib and hips. The ovaries were then surgically detached, and the skin was closed. Subsequently, OVX mice were euthanised at 12 weeks (OVX‐12W) and 18 weeks (OVX‐18W) for further analysis.

Oestrogen treatment involved daily subcutaneous injections of 1 µg 17‐β‐estradiol‐3‐benzoate (Sigma‐Aldrich, cat#E8515).^34^

### 2.3 Serum estradiol level assay

Blood was obtained from mice with or without OVX. Serum was separated by centrifuging the blood at 2000 × *g* for 10 min at 4°C. Estradiol levels were determined using the Estradiol ELISA Kit (Beyotime, cat#PE223). Briefly, 90 µL of serum and standard samples were pipetted into each well of a plate, followed by 10 µL of horseradish peroxidase (HRP)‐conjugated estradiol to each, respectively. The samples were mixed for 10 s and incubated at ambient temperature, shielded from light, for 120 min. After washing the wells three times, 100 µL of 3,3′,5,5′‐Tetramethylbenzidine (TMB) substrate solution was introduced into each well. The plate was then incubated at ambient temperature, shielded from light, for 15–20 min. The reaction was subsequently terminated by stop solution. After detecting absorbance at 450 nm, the serum estradiol levels were calculated based on the standard curve.

### 2.4 Isolation of muscle FAPs

Primary FAPs were isolated as previously reported.^35^, ^36^ Supraspinatus muscle tissue firstly underwent initial fine dissection, followed by a 1‐h digestion with Collagenase II (700–800 U/mL, Worthington Biochemical, cat#LS004177). Subsequently, it underwent a 30‐min digestion with a mixture of Collagenase II and Dispase II (11 U/mL, Life Technologies, cat#17105‐041). The digested suspension was then passed through a 40 µm cell strainer (BD Falcon, cat#352340) to remove debris. Adaptations for cell preparation for flow cytometry were implemented based on established protocols.^9^, ^18^, ^35^, ^36^, ^37^, ^38^, ^39^ Fluorescence‐activated cell sorting (FACS) was performed on a BD Influx sorter to isolate the desired FAP populations. Mouse FAPs were identified as CD45−/CD31−/PDGFRα+ cells, while human FAPs were gated as CD31−/CD45−/CD56−/CD34+ cells.

### 2.5 Cell culture, adipogenic differentiation and treatment

Primary FAPs were maintained in a growth medium composed of α‐MEM (Cellgro, cat#10‐022‐CV) with 20% fetal bovine serum (FBS; Yeasen, cat#40131ES76) and 1% penicillin–streptomycin (Gibco, cat#15140‐122) at 37°C in a 5% CO_2_ environment. As previously described,^9^ adipogenic differentiation was induced by switching to adipogenic differentiation medium (ADM). For functional experiments, FAPs were treated with 100 nM 17β‐estradiol (E2, MCE, cat#HY‐B0141), 50 µM ESR1‐specific agonist propyl pyrazole triol (PPT, MCE, cat#263717‐53‐9) or 10 µM ESR1 inhibitor MPP dihydrochloride (MPP, MCE, cat#911295‐24‐4).

### 2.6 Immunohistology and immunofluorescent staining

Cryosections (10 µm thick) of fresh muscle tissues or cells were fixed with 4% Paraformaldehyde (PFA) for 15 min at ambient temperature to preserve structural integrity. After fixation, the samples were incubated with .5% Triton X‐100 for 15 min at ambient temperature to allow antibody access to intracellular structures. After that, blocking was performed by incubating samples in phosphate‐buffered saline (PBS) containing 1% bovine serum albumin (BSA; Beyotime, cat#ST023). After blocking, the samples were incubated at 4°C overnight with primary antibodies, including Laminin (Abcam, cat#ab44941), PDGFRα (Abcam, cat#ab203491), PPARγ (Abcam, cat#ab178860) and perilipin A/B (Millipore, cat#P1873). Appropriate Alexa Fluor‐conjugated secondary (Invitrogen) were applied at ambient temperature for 1 h. The nuclei were labelled with DAPI (Vector Laboratories, cat#H‐1200) and mounted using an anti‐fade reagent (Vector Laboratories, cat#H‐100). Subsequent image analysis was performed using ImageJ software.

### 2.7 Oil Red O staining

To assess the ability of adipogenesis, 4% PFA was firstly used to fix differentiated FAPs. Subsequently, the samples underwent a 15‐min permeabilisation using .5% Triton X‐100 at room temperature, followed by a 10‐min staining with Oil Red O (Solarbio, cat#G1260). Nuclear staining was then carried out using DAPI. Images were captured from three random fields per sample, and subsequent analyses were performed. Specifically, the area of Oil Red O staining was quantified, and the number of nuclei was counted using ImageJ software. The results were presented as the ratio of area to nuclear count.

### 2.8 Triglycerides quantification analysis

For assessing triglyceride levels, muscle samples homogenised in isopropanol were analysed using a triglycerides quantification kit (Elabscience, cat#E‐BC‐K261‐M). After centrifuging at 10 000 × *g* for 3 min. The supernatant was harvested, and absorbance was quantified at 510 nm.

### 2.9 Gene expression analysis

RNA was extracted using TRIzol (Invitrogen, cat#15596‐018) and reverse‐transcribed with MuLV reverse transcriptase (NEB, cat#M0253L). RT‐qPCR employed SYBR Green qPCR Mix (ABclonal, cat#RK21203). The RT‐qPCR primers are listed in Table S2.

### 2.10 Western blotting

Protein lysis was performed on ice using a mixture of RIPA lysis buffer (Beyotime, cat#P0013B), protease inhibitor (Beyotime, cat#P1008) and phosphatase inhibitor (Beyotime, cat#P1082). The Bicinchoninic Acid Assay (BCA) protein assay kit (Beyotime, cat#P0010S) was used to quantify protein concentrations. Heat denaturation was then performed for the extracts for 10 min with the addition of sample loading buffer (Yeasen, cat#20315ES05) at 95°C. Then proteins were separated via sodium dodecyl sulphate‐polyacrylamide gel electrophoresis (SDS‐PAGE). Next, proteins were transferred to polyvinylidene fluoride (PVDF) membranes and blocked with a 5% BSA solution in Tris Buffered Saline with Tween 20 (TBST) for 1 h. Overnight incubation of the membranes was carried out at 4°C on a shaker with primary antibodies diluted in TBST containing 1% BSA, including ESR1 (Abcam, cat#ab32063), METTL3 (Abcam, cat#ab195352), PPARγ (Proteintech, cat#66936‐1‐Ig) and glyceraldehyde‐3‐phosphate dehydrogenase (GAPDH) (CST, cat#2118L). Next, the blots were treated with the corresponding secondary antibodies (Santa Cruz, cat#sc‐2005 and Santa Cruz, cat#sc‐2357). Protein bands were then visualised using chemiluminescence and analysed with ImageJ. To display all proteins on a single membrane, the blot was stripped using rapid antibody stripping buffer (Epizyme, cat#PS107) and re‐probed with another primary antibody.

### 2.11 Gene overexpression

The overexpression of ESR1 in human FAPs was achieved through adenovirus infection. The ESR1 cDNA was first inserted into the Sal I and EcoRV sites of the pAdTrack‐Cytomegalovirus (CMV) adenoviral vector (Addgene, cat#16405). The vectors were then linearised using PmeI and introduced into BJ5183‐AD‐1 cells. Recombinant adenoviral plasmids were applied for transfection into 293 cells for propagation and purification using Lipofectamine 2000 (Thermo Fisher, cat#11668019). A viral concentration of 1.2 × 10^6^ units/µL was applied to infect 3 × 10^5^ human FAPs. Various assays were performed on these cells 48 h after infection.

### 2.12 m6A dot blot assay

To disrupt the secondary structure, mRNA samples were subjected to 3 min of heating at 95°C, followed by rapid cooling on ice for 3 min. Nitrocellulose membranes were prepared with circular imprints created using the blunt end of a 1 mL pipette tip. Then, aliquots of mRNA were applied to the membranes, air‐dried at room temperature for 5 min and cross‐linked under UV light for 1 h. The membranes were blocked for 1 h with 5% BSA in TBS. Primary incubation was carried out overnight at 4°C using anti‐m6A antibody (Abcam, cat#151230). Chemiluminescence was used to visualise global m6A levels, which were subsequently quantified with ImageJ.

### 2.13 Methylated RNA immunoprecipitation‐qPCR (MeRIP‐qPCR) and MeRIP‐sequencing (MeRIP‐seq)

MeRIP was conducted using the m6A RNA Enrichment Kit (Epigentek, cat#P‐9018‐48). Total RNA (20 µg) was immunoprecipitated with 1/10 reserved as the input control (Input). Immunocapture involved mRNA samples, m6A antibodies, affinity beads and buffer vortexed for 90 min at room temperature. RNA fragmentation was achieved using cleavage enzymes, followed by proteinase K treatment and RNA purification to isolate m6A‐enriched RNA. The immunoprecipitated m6A RNA (IP) was then subjected to RT‐qPCR analysis, with primers provided in Table S3.

For MeRIP‐seq, concentrated m6A RNA fragments were prepared into sequencing libraries using the Stranded mRNA‐seq Kit (Illumina). Libraries from input and enriched samples were sequenced on an Illumina HiSeq 4000 platform. Differentially enriched m6A peaks were identified with exomePeak (false discovery rate (FDR) ≤ .05) and visualised using IGV software.

### 2.14 Dual luciferase reporter assay

To evaluate the impact of the mutant of 717 bp site in Esr1, the pmirGLO‐Esr1‐wild type (WT) and pmirGLO‐Esr1‐Mut plasmids were constructed based on pmirGLO vector (MIAOLING BIOLOGY, cat#P0198). The WT or mutant of Coding DNA Sequence (CDS) of Esr1 was cloned behind the firefly luciferase (F‐luc) coding region. Then they were transfected into FAPs from Esr1 KO and Esr1 f/f mice. Subsequently, the activities of F‐luc and Renilla luciferase (R‐luc) were assessed using the Dual Luciferase Reporter Assay Kit (YEASEN, cat#11402ES60).

To illustrate the role of ESR1 in regulating the transcription of PPARγ and METTL3, WT or mutant PPARγ promoter containing ESR1 responsive elements were ligated into the pGL3‐basic luciferase reporter construct (Promega). Then human FAPs were co‐transfected with luciferase reporter constructs and the pCMV‐ESR1 expression vector (MIAOLING BIOLOGY, cat#P58644) using Lipofectamine 2000 (Thermo Fisher, cat#11668019). Briefly, Lipofectamine 2000 and plasmids were diluted in reduced serum medium (Thermo Fisher, cat#31985062) at ambient temperature for 5 min. After mixing, the two components were incubated at ambient temperature for 20 min before being added to FAPs. We harvested cells 48 h post‐transfection and measured F‐luc and R‐luc activities

### 2.15 RNA stability assay

FAPs were exposed to 5 µg/mL actinomycin D (Act‐D; MCE, cat#HY‐17559) to inhibit transcription. Cells were harvested at 0, 3, 6, 9 and 12 h for RNA extraction. Degradation of target RNA was detected via RT‐qPCR, with GAPDH as the reference gene.

### 2.16 Protein stability assay

Cycloheximide (CHX, MCE, cat#HY‐12302, 100 µg/mL) was applied to evaluate protein stability in FAPs. After 0, 3, 6, 9 and 12 h of treatment, FAPs were harvested, and protein levels were evaluated through western blot.

### 2.17 Chromatin immunoprecipitation‐qPCR assay

The chromatin immunoprecipitation (ChIP) experiment was conducted with the SimpleChIP Plus Kit (CST, cat#9005S). In brief, FAPs were fixed in 1% formaldehyde for 10 min, then lysed and sonicated. Immunoprecipitation employed antibodies against ESR1 (Abcam, cat#ab32063) or rabbit IgG (CST, cat#2729). After immunoprecipitation, the DNA fragments were quantified by ChIP‐qPCR. Primers used for detecting ESR1 binding sites on the PPARγ and METTL3 promoters were listed in Table S4.

### 2.18 Gait analysis

According to established methods,^40^, ^41^ stride length and paw surface area were recorded using the Noldus Catwalk system to evaluate muscle strength and performance.^42^, ^43^ The mice moved freely in an 85‐cm‐long and 8.5‐cm‐wide corridor, with their movements were documented by tracking footprints with consistent trajectories.

### 2.19 Treadmill test

Mice underwent adaptation training for 2 days before formal testing on a ZII‐PT/5S treadmill. The exhaustion test began at 10 m/min on a 15‐degree incline, with speed increasing by 2 m/min every 2 min up to 20 m/min. Exhaustion was defined when the mice were unable to run despite three manual prompts and exhibited specific behaviours in the novel environment. The duration and distance of the run were then systematically recorded for further analysis.

### 2.20 Bulk RNA‐seq and analysis

Following the acquisition of purified mRNA, RNA sequencing libraries were constructed using the RNA Library Prep Kit (New England Biolabs, cat#E7530L). Next, a cDNA library with an average insert size of 300 bp was prepared using a non‐stranded method. Paired‐end sequencing was performed on a NovaSeq 6000 sequencer with a 2×150 bp read length. SeqPrep and Sickle websites were used for quality control.

Next, HISAT2 was employed to align the clean paired‐end reads to the GRCh38.98 reference genome, and gene abundances were quantified with RSEM.^44^ DEGseq was used for differential expression analysis to identify significantly differentially expressed genes (*p* < .05).^45^ For further analysis, Gene Ontology (GO) and Kyoto Encyclopedia of Genes and Genomes (KEGG) analyses were performed using Goatools and KOBAS, respectively.^46^

### 2.21 Statistical analysis

Student's *t*‐tests, one‐way analysis of variance (ANOVA) and two‐way ANOVA were used for statistical analysis using GraphPad Prism 9 (GraphPad). The results are presented as mean ± standard deviation, with statistical significance defined as *p* < .05. Experiments included three biological replicates and three technical replicates conducted blindly.

## 3 RESULTS

### 3.1 Decreased expression of ESR1/METTL3 and increased adipogenesis ability in FAPs was found in post‐menopausal female

**FIGURE 1:**
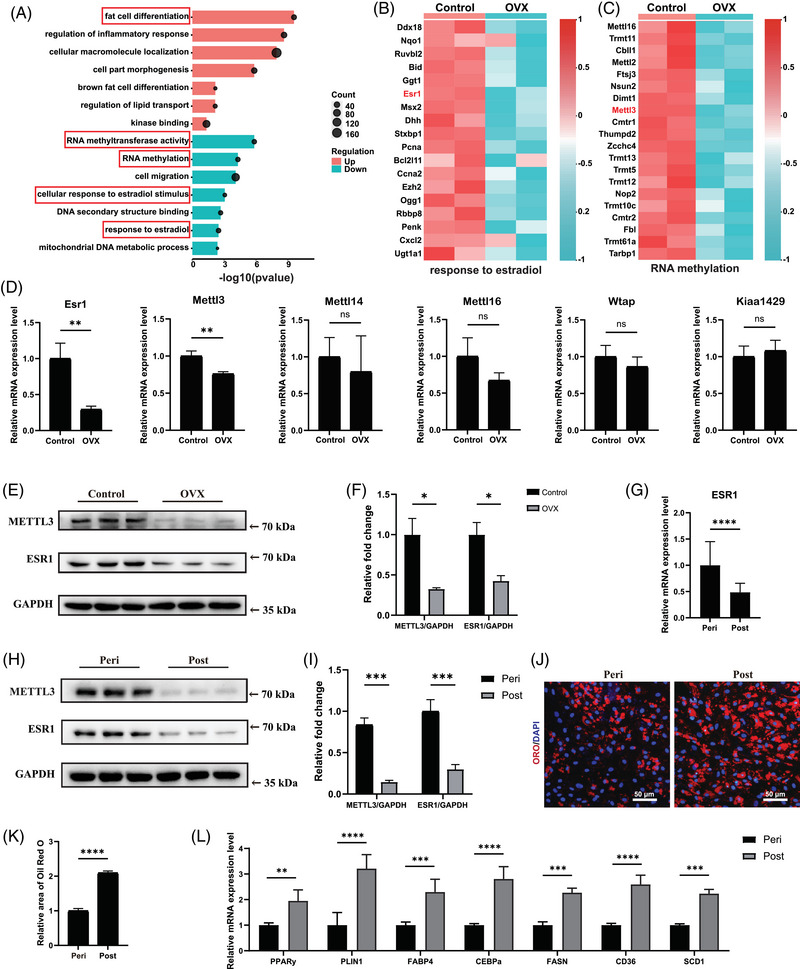
Decreased expression of oestrogen receptor 1 (ESR1)/methyltransferase‐like 3 (METTL3) and increased adipogenesis ability in fibro/adipogenic progenitors (FAPs) was found in post‐menopausal female. (A) Bubble chart of GO analysis of up‐regulated and down‐regulated genes in FAPs from sham mouse (Control) and FAPs from ovariectomy (OVX) mouse. Key GO enrichment terms were highlighted with red frames. (B) Heat map of differentially expressed genes associated with the GO term of ESR1 signalling in FAPs from sham mouse (Control) and FAPs from OVX mouse. (C) Heat map of differentially expressed genes associated with the GO term of RNA methylation in FAPs from sham mouse (Control) and FAPs from OVX mouse. (D) Relative mRNA expression of Esr1 and N6‐methyladenosine (m6A) methyltransferases in FAPs from sham mouse (Control) and FAPs from OVX mouse (*n* = 3 mice/group). (E, F) Protein levels and quantitative assessment of ESR1, METTL3 and GAPDH of FAPs from sham mouse (Control) and FAPs from OVX mouse (*n* = 3 mice/group). (G) Relative mRNA expression of ESR1 in FAPs from peri‐menopausal females (Peri) and FAPs from post‐menopausal females (Post) (*n* = 8 patients/group). (H, I) Protein levels and quantitative assessment of ESR1, METTL3 and GAPDH in FAPs from peri‐menopausal females (Peri) and FAPs from post‐menopausal females (Post) (*n* = 3 patients/group). (J, K) Oil Red O staining and quantitative assessment of lipid accumulation in FAPs from peri‐menopausal females (Peri) and FAPs from post‐menopausal females (Post) after adipogenic differentiation for 10 days (*n* = 5 per condition). Red indicated Oil Red O, blue indicated DAPI and the merged images were shown. Scale bar, 50 µm. (L) Relative mRNA expression of adipogenic and lipogenic genes in FAPs from peri‐menopausal females (Peri) and FAPs from post‐menopausal females (Post) after adipogenic differentiation for 10 days (*n* = 3 per condition). Data were shown as mean ± standard deviation (SD), ns indicated no significant differences, * indicated *p* < .05, ** indicated *p* < .01, *** indicated *p* < .001, **** indicated *p* < .0001.

To investigate the biological effects of oestrogen deficiency in muscular fatty infiltration, supraspinatus muscles were first obtained from mice with and without OVX. Serum estradiol levels in OVX mice were significantly decreased than those in the control group (Figure S1A). There was more severe muscular fat accumulation in supraspinatus samples from OVX mice, which got worse with the duration of OVX, as supported by immunofluorescent staining of PLIN1 (Figure S1B,C). Consistently, OVX mice exhibited elevated muscular triglyceride level in supraspinatus muscles (Figure S1D). Thus, significant decreased oestrogen contributed to excessive muscular fatty infiltration of supraspinatus muscles. To investigate the detailed mechanism of it, we first isolated fresh FAPs from mice with or without OVX by FACS, and confirmed their identity through staining with the FAP‐specific marker PDGFRα (Figure S1E,F). RNA sequencing was performed to assess the transcriptomic profiles of freshly isolated FAPs from mice with or without OVX. There were 2560 up‐regulated genes and 3990 down‐regulated genes among the differentially expressed genes in OVX‐FAPs compared with the control group (Figure S1G). GO analysis of the differentially expressed genes indicated that ESR signalling and RNA methylation were decreased, while fat cell differentiation was increased in OVX‐FAPs (Figure 1A). Especially, the key m6a methyltransferase gene Mettl3 was identified in GO term RNA methylation, and Esr1 was found as a differentially expressed gene in GO term ESR signalling (Figure 1B,C). Consistently, the RT‐qPCR results demonstrated that Esr1 and Mettl3 significantly reduced in OVX‐FAPs, with no significant differences in other RNA methyltransferases (Figure 1D). Immunoblotting further confirmed the decreased levels of ESR1 and METTL3 in OVX‐FAPs (Figure 1E,F). Since fat cell differentiation was enriched among the up‐regulated genes in the GO analysis, RT‐qPCR was performed to assess key adipogenic and lipogenic genes, including Pparγ, Plin1, Fabp4, C/ebpα, Fasn, Cd36 and Scd1. The results showed a significant increase in mRNA expression levels in OVX‐FAPs, which got worse with the duration of oestrogen deficiency (Figure S1H). Combined, these data indicated that there were decreased Mettl3 and Esr1 expression levels, along with enhanced adipogenesis potential of FAPs in OVX mice.

Then samples from patients were collected to confirm the aforementioned findings in post‐menopausal females. No notable variations were found in clinical phenotypes, including age, body mass index (BMI) and glucose levels, while serum estradiol levels differed significantly between peri‐menopausal and post‐menopausal women (Table S1). There was also more severe muscular fat accumulation in supraspinatus samples from post‐menopausal female patients, supported by immunofluorescent staining of PLIN1 and triglyceride level measurement (Figure S2A–C). For subsequent analyses, fresh FAPs from peri‐menopausal women and post‐menopausal women were isolated using FACS and verified through staining with the FAP‐specific marker PDGFRα (Figure S2D,E). RT‐qPCR and immunoblotting results suggested that expression levels of ESR1 and METTL3 were significantly down‐regulated during menopause progression (Figure 1G–I). To evaluate the adipogenic differentiation ability, adipogenic induction of FAPs from both peri‐menopausal and post‐menopausal female patients were performed. Given that the transcriptional profile may be altered after adipogenic differentiation, we also performed immunoblotting to evaluate the expression of METTL3 and ESR1. The results indicated a similar trend to that observed in peri‐menopausal FAPs before adipogenesis (Figure S2F,G). The Oil Red O staining of differentiated cells revealed significant increased lipid droplets in post‐menopausal female FAPs (Figure 1J,K). In consistent, RT‐qPCR analysis also revealed significantly increased expression of adipogenic and lipogenic genes PPARγ, PLIN1, FABP4, C/EBPα, FASN, CD36 and SCD1 indicating elevated adipogenic differentiation ability of post‐menopausal female FAPs (Figure 1L).

Combined, these results highlighted the down‐regulated expression of ESR1 and METTL3, along with an increased adipogenic differentiation ability in post‐menopausal females.

### 3.2 METTL3‐mediated m6A methylation promoted ESR1 mRNA stability at the post‐transcriptional level in FAPs

**FIGURE 2:**
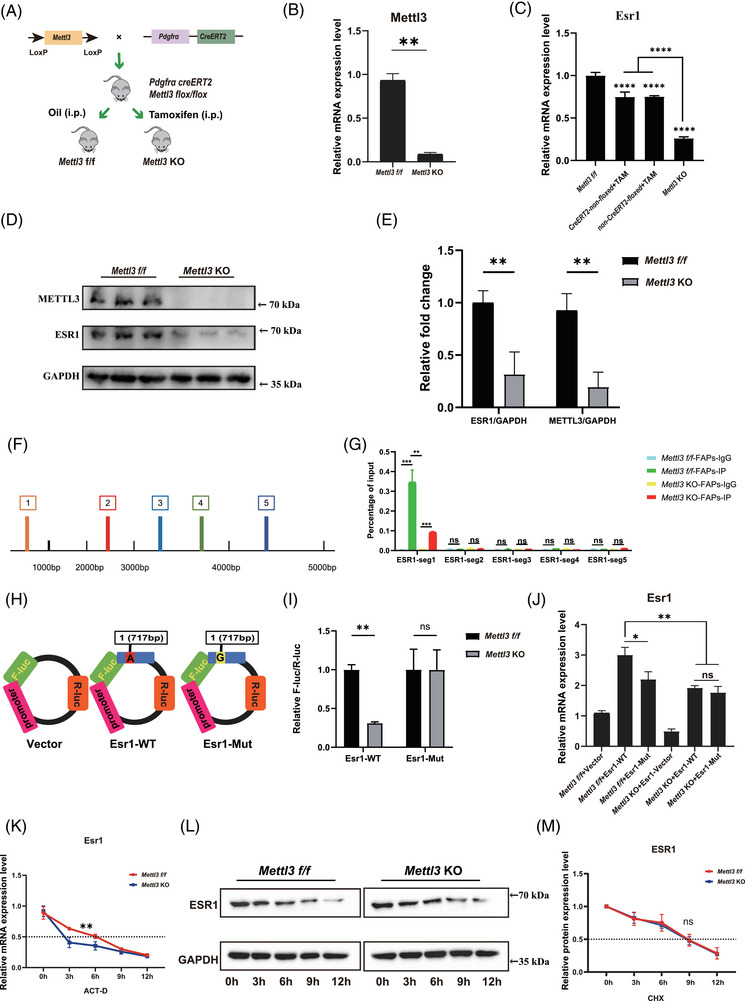
Methyltransferase‐like 3 (METTL3)‐mediated N6‐methyladenosine (m6A) methylation promoted oestrogen receptor 1 (ESR1) mRNA stability at the post‐transcriptional level in fibro/adipogenic progenitors (FAPs). (A) Scheme of the generation of FAPs specific Mettl3 flox/flox (Mettl3 f/f) mouse and FAPs‐specific Mettl3 knockout (Mettl3 KO) mouse. (B) Relative mRNA expression of Mettl3 in FAPs from Mettl3 f/f mice and Mettl3 KO mice (*n* = 3 mice/group). (C) Relative mRNA expression of Esr1 in FAPs from Pdgfrα‐CreERT2; Mettl3 flox/flox with no tamoxifen (TAM) treatment (Mettl3 f/f), Pdgfrα‐non‐CreERT2; Mettl3 flox/flox with TAM treatment (non‐CreERT2‐floxed + TAM), Pdgfrα‐non‐CreERT2; Mettl3 +/+ with TAM treatment (CreERT2‐non‐floxed + TAM) and Pdgfrα‐CreERT2; Mettl3 flox/flox with TAM treatment (Mettl3 KO) (*n* = 3 mice/group). (D, E) Protein expression and quantitative assessment of METTL3, ESR1 and GAPDH in freshly isolated FAPs from Mettl3 f/f mice and Mettl3 KO mice (*n* = 3 mice/group). (F) The potential m6A modification sites on Esr1 mRNA predicted by JASPAR. The Esr1 mRNA sequence was divided into five segments based on these predicted m6A methylation sites. (G) MeRIP‐qPCR analysis of the five segments of Esr1 between the anti‐IgG (IgG) group and anti‐m6A (IP) group in FAPs from Mettl3 f/f mice and Mettl3 KO mice (*n* = 3 per condition). (H) Schematic representation of Vector, Esr1 wild type (Esr1‐WT) and Esr1 mutant (Esr1‐Mut) luciferase reporter constructs. (I) Luciferase activity in FAPs from Mettl3 f/f mice and Mettl3 KO mice 48 h post‐transfection (*n* = 3 per condition). (J) Relative mRNA expression of Esr1 in FAPs from Mettl3 f/f mice and Mettl3 KO mice after transfection with pcDNA‐Esr1‐WT or pcDNA‐Esr1‐Mut for 48 h (*n* = 3 per condition). (K) RNA stability assay of Esr1 mRNA. Actinomycin D (Act‐D) was used in FAPs from Mettl3 f/f mice and Mettl3 KO mice, and ESR1 mRNA expression in FAPs from Mettl3 f/f mice and Mettl3 KO mice was analysed at indicated times (*n* = 3 per condition). (L, M) Protein stability assay of ESR1. Cycloheximide (CHX) was first used in FAPs from Mettl3 f/f mice and Mettl3 KO mice, and ESR1 protein expression was analysed at indicated times (*n* = 3 per condition). Data were shown as mean ± standard deviation (SD), ns indicated no significant differences, * indicated *p* < .05, ** indicated *p* < .01, *** indicated *p* < .001, **** indicated *p* < .0001.

Previous studies indicated potential association between ESR1 and epigenetic changes in post‐menopause.^19^, ^20^ Since m6A modification is a crucial RNA epigenetic modification and METTL3 is a key m6a methyltransferase gene, we hypothesised that decreased ESR1 could be driven by METTL3‐mediated m6A modification in FAPs. We subsequently generated FAP‐specific Mettl3 KO mice by administering i.p. TAM injections in Pdgfrα‐CreERT2; Mettl3 flox/flox (Mettl3 f/f) mice every other day for 1 week, starting at 3 weeks of age and continuing until 4 weeks of age (Figure 2A). Then fresh FAPs were isolated for subsequent assays. A significant decrease in Mettl3 expression was detected in Mettl3 KO FAPs (Figure 2B,D,E). Similarly, the overall m6A modification level also markedly decreased in Mettl3 KO FAPs (Figure S3A,B). The RT‐qPCR and Immunoblotting assays further revealed significantly decreased expression of Esr1 in Mettl3 KO FAPs (Figure 2C–E). Combined, these results indicated the expression of Esr1 could be regulated by Mettl3.

Considering the pivotal role of Mettl3 in m6A modification, we next explored if Mettl3 regulated expression of Esr1 by m6A modification. RNA methylation site predictor (SRAMP) was utilised to predict m6A methylation sites on Esr1, revealing five candidate sites of m6A methylation in Esr1 sequence (Figure S4A). Thus, five primer pairs were designed to explore functional m6A methylation site (Figure 2F). MeRIP‐qPCR analysis indicated the m6A methylation level was significantly decreased after KO of Mettl3 only in the first potential m6A site at position 717 bp of Esr1 mRNA (Figure 2G). A luciferase reporter assay was subsequently conducted using WT (Esr1 717A) and m6A site mutant (Mut) (Esr1 717G) plasmids (Figure 2H). The Esr1 717A site was mutated to Esr1 717G, resulting in a synonymous mutation. There was a significant increase of relative luciferase intensity in Mettl3 f/f FAPs following transfection with Esr1‐WT plasmids when compared those in Mettl3 KO FAPs (Figure 2I). In contrast, no statistically difference of relative luciferase intensity was found between Mettl3 f/f and Mettl3 KO FAPs after transfected with Esr1‐Mut plasmids (Figure 2I). The results demonstrated that the first potential m6A site (717 bp) effectively regulated Esr1 m6A methylation levels. Furthermore, MeRIP‐seq was performed on control, OVX, Mettl3 f/f and Mettl3 KO FAPs. The results suggested a significant reduction in m6A enrichment on chromosome 10 (positions 4 712 223–4 712 792) in OVX compared to control FAPs, as well as in Mettl3 KO compared to Mettl3 f/f FAPs. This region also included the potential m6A modification site at position 717 bp A (Figure S4B). Therefore, MeRIP‐seq analysis further strengthened the results of our study that Mettl3‐mediaed m6A methylation could modify Esr1 mRNA.

To evaluate the effects of METTL3‐mediated m6A modification on ESR1 expression, we established WT and mutant (Mut) Esr1 plasmids targeting the Esr1 717 bp mutation site. These plasmids were then transfected into Mettl3 f/f FAPs and Mettl3 KO FAPs. The results demonstrated that mutating this site significantly reduced Esr1 mRNA in Mettl3 f/f FAPs (Figure 2J). Notably, KO of Mettl3 decreased Esr1 mRNA expression regardless of the mutation status at Esr1 717A (Figure 2J). Furthermore, even with transfection of Esr1‐WT plasmid, reduced Esr1 mRNA expression was observed in Mettl3 KO FAPs (Figure 2J). Collectively, these results suggested that Mettl3‐mediated m6A modification increased Esr1 mRNA expression via the Esr1 717 bp site.

To further investigate the impact of METTL3‐mediated m6A modification on ESR1 expression, we conducted both RNA and protein stability experiments. Mettl3 f/f FAPs and Mettl3 KO FAPs were exposed to Act‐D to block transcription or CHX to inhibit translation. The analysis revealed a notably reduced half‐life of Esr1 mRNA in Mettl3 KO FAPs (Figure 2K), highlighting the critical role of METTL3 in maintaining Esr1 mRNA stability. In contrast, the half‐life of ESR1 protein remained unchanged between Mettl3 f/f FAPs and Mettl3 KO FAPs, as assessed by immunoblotting (Figure 2L,M). These results collectively suggest that METTL3‐mediated m6A modification exerts its regulatory effects on ESR1 primarily through modulation of mRNA stability at the post‐transcriptional level in FAPs.

### 3.3 ESR1 could suppress adipogenesis ability of FAPs

**FIGURE 3:**
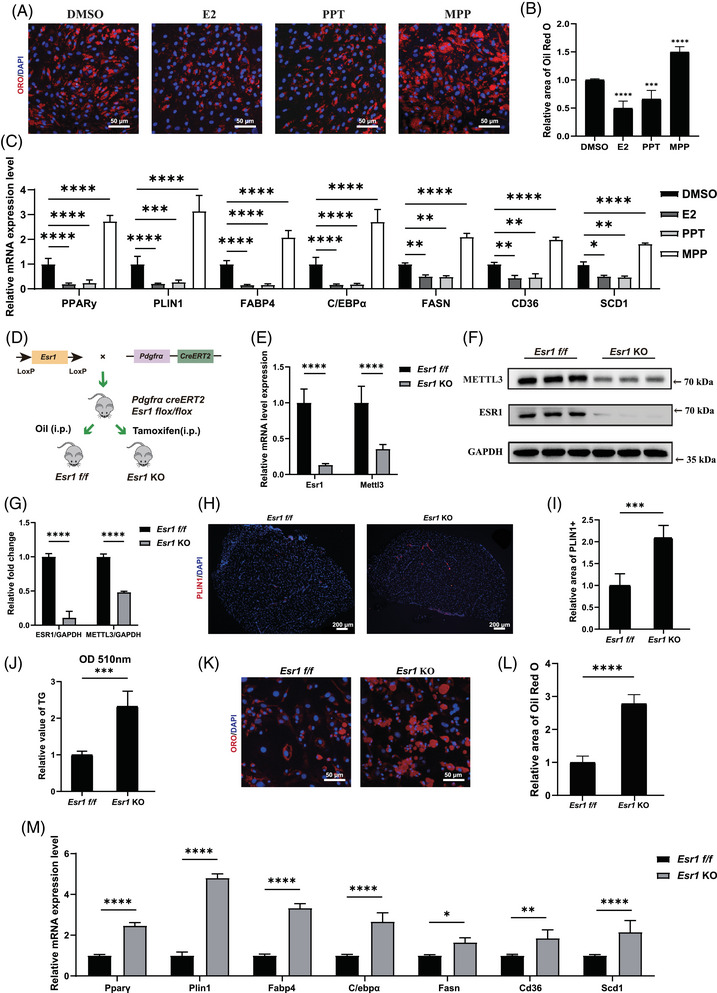
Oestrogen receptor 1 (ESR1) could suppress adipogenesis ability of fibro/adipogenic progenitors (FAPs). (A, B) Oil Red O staining and quantitative assessment of lipid droplets formation in human FAPs following 10 days of adipogenic differentiation with treatment by dimethyl sulfoxide (DMSO), oestrogen (E2), ESR1 agonist propyl pyrazole triol (PPT) or ESR1 inhibitor MPP (MPP), respectively (*n* = 5 per condition). Red indicated Oil Red O, blue indicated DAPI and the merged images were shown. Scale bar, 50 µm. (C) Relative mRNA expression of adipogenic genes in human FAPs following 10 days of adipogenic differentiation with treatment by DMSO, oestrogen (E2), ESR1 agonist PPT (PPT) or ESR1 inhibitor MPP (MPP), respectively (*n* = 3 per condition). (D) Scheme of the generation of FAPs‐specific Esr1 flox/flox (Esr1 f/f) mouse and Esr1 knockout (Esr1 KO) mouse. (E) Relative mRNA expression of Esr1 and Mettl3 between FAPs from Esr1 f/f mice and Esr1 KO mice (*n* = 3 mice/group). (F, G) Protein levels and quantitative assessment of ESR1, methyltransferase‐like 3 (METTL3) and GAPDH between FAPs from Esr1 f/f mice and Esr1 KO mice (*n* = 3 mice/group). (H, I) Immunofluorescence staining of PLIN1 and quantitative assessment of lipid droplets in the supraspinatus muscles between Esr1 f/f mice and Esr1 KO mice (*n* = 4 mice/group). Red indicated PLIN1, blue indicated DAPI and the merged images were shown. Scale bar, 200 µm. (J) Quantification measurement of triglycerides in the supraspinatus muscle of Esr1 f/f mice and Esr1 KO mice (*n* = 5 mice/group). (K, L) Oil Red O staining and quantitative assessment of lipid droplets formation in FAPs from Esr1 f/f mice and Esr1 KO mice following 10 days of adipogenic differentiation (*n* = 5 per condition). Red indicated Oil Red O, blue indicated DAPI and the merged images were shown. Scale bar, 50 µm. (M) Relative mRNA expression of adipogenic and lipogenic genes between FAPs from Esr1 f/f mice and Esr1 KO mice following 10 days of adipogenic differentiation (*n* = 3 per condition). Data were shown as mean ± standard deviation (SD), * indicated *p* < .05, ** indicated *p* < .01, *** indicated *p* < .001, **** indicated *p* < .0001.

Previous studies have proved that ESR1 functioned as a critical role in adipogenesis of adipocytes, hepatocytes and bone marrow‐derived mesenchymal stem cells.^26^, ^47^, ^48^, ^49^, ^50^, ^51^ Thus, we investigated the role of ESR1 in adipogenic differentiation of FAPs. Female primary FAPs were cultured in ADM with treatments of oestrogen (E2), ESR1 agonist PPT or ESR1 inhibitor MPP, respectively. After 10‐day adipogenic induction, Oil Red O staining demonstrated reduced lipid formation with ESR1 agonist PPT or E2 treatment, whereas ESR1 antagonist MPP increased fatty infiltration (Figure 3A,B). Consistently, RT‐qPCR results confirmed down‐regulated adipogenic and lipogenic genes in FAPs under treatment with E2 and PPT, while MPP up‐regulated adipogenic and lipogenic genes (Figure 3C). These findings suggested that activation of ESR1 signalling inhibited the adipogenic potential of FAPs.

To further illustrate the role of ESR1 in adipogenesis of FAPs, FAPs‐specific Esr1 KO mice were generated by i.p. TAM injection in Pdgfrα‐CreERT2; Esr1 flox/flox (Esr1 f/f) mice every 2 days for 1 week (from 3‐week‐old to 4‐week‐old) (Figure 3D). RT‐qPCR and western blot confirmed successful KO effect of Esr1 in FAPs (Figure 3E–G). Immunohistochemistry and triglyceride quantification subsequently demonstrated significantly more fat accumulation in the supraspinatus muscle of 12‐week‐old Esr1 KO mice (Figure 3H–J). Immunofluorescence staining for PPARγ was conducted on freshly isolated Esr1 KO and Esr1 f/f FAPs. Esr1 KO FAPs exhibited a higher proportion of PPARγ‐positive cells, suggesting an increased adipogenic potential (Figure S5A,B). Esr1 KO and Esr1 f/f FAPs were then supplemented with ADM. After 10 days of adipogenic induction, Oil Red O staining and RT‐qPCR were conducted to evaluate adipogenic differentiation ability. There was an elevated accumulation of lipid content and increased expression of adipogenic‐related genes in Esr1 KO FAPs, revealing that the loss of ESR1 exacerbated adipogenic potential of FAPs (Figure 3K–M). Combined, these data demonstrated that ESR1 could suppress adipogenesis of FAPs.

### 3.4 ESR1 suppressed adipogenic differentiation of FAPs by inhibiting transcription of PPARγ

**FIGURE 4:**
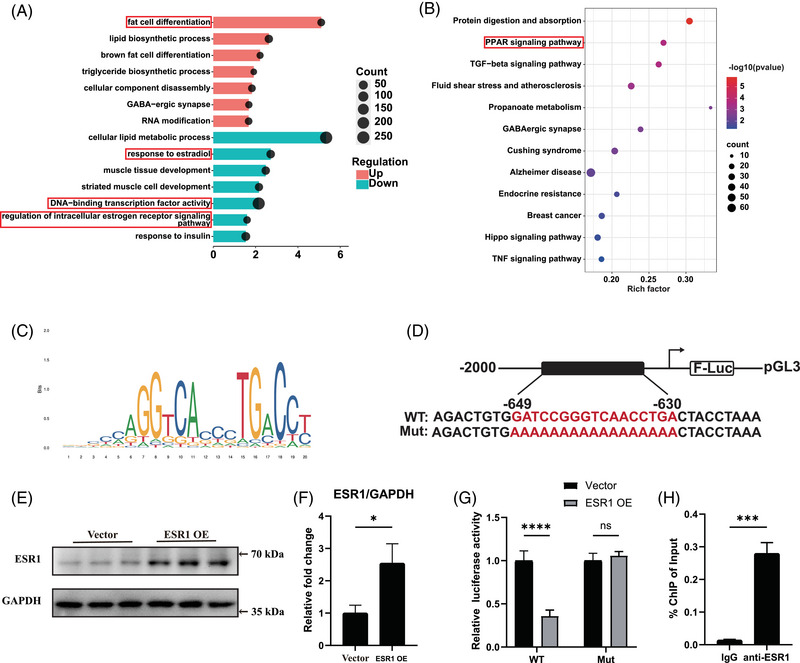
Oestrogen receptor 1 (ESR1) suppressed adipogenic differentiation of fibro/adipogenic progenitors (FAPs) by inhibiting transcription of peroxisome proliferator‐activated receptor gamma (PPARγ). (A) Bubble chart of GO analysis of up‐regulated and down‐regulated genes in FAPs from Esr1 knockout (KO) mouse when compared with those from Esr1 f/f mouse. Key GO terms were highlighted with red frames. (B) KEGG analysis of up‐regulated genes in FAPs from Esr1 KO mouse compared to those from Esr1 f/f mice. Key KEGG terms were highlighted with a red frame. (C) The sequence logo of potential ESR1 binding sites on the PPARγ promoter was predicted using JASPAR. (D) Scheme of construction of wild type (WT) and mutant pGL3‐PPARγ promoter reporter plasmids. (E, F) Protein levels and quantitative assessment of ESR1 and GAPDH after transfection with ESR1 overexpression (ESR1 OE) and vector (Vector) plasmids (*n* = 3 per condition). (G) Quantitative assessment of luciferase activity after transfection with WT and mutant (Mut) pGL3‐PPARγ promoter reporter plasmids in female FAPs transfected with ESR1 overexpression plasmid (*n* = 3 per condition). (H) Chromatin immunoprecipitation (ChIP)‐qPCR analysis for ESR1 binding to the PPARγ promoter in female FAPs under incubation with IgG or anti‐ESR1 antibodies (*n* = 3 per condition). Data were shown as mean ± standard deviation (SD), ns indicated no significant differences, * indicated *p* < .05, *** indicated *p* < .001, **** indicated *p* < .0001.

We next performed RNA‐seq of Esr1 KO and *Esr1 f/f* FAPs to explore the underlying molecular mechanism of how ESR1 regulated adipogenic differentiation of FAPs. The volcano plot demonstrated that there were 3111 up‐regulated genes and 3929 down‐regulated genes in Esr1 KO compared to Esr1 f/f FAPs (Figure S5C). GO analysis of the genes up‐regulated in Esr1 KO FAPs revealed the activation of critical terms related to fat cell differentiation, whereas suppressed ESR1‐related signalling and transcription factor signalling were enriched in the down‐regulated GO terms of Esr1 KO FAPs (Figure 4A). Furthermore, KEGG analysis suggested PPAR signalling, the key item of lipid metabolism, was up‐regulated in Esr1 KO FAPs (Figure 4B). Notably, PPARγ signalling is the most critical pathway for adipogenesis within the PPAR signalling pathway.^52^ Since ESR1 also functions as a transcription factor activated by estrogen,^29^, ^30^ these data indicated ESR1 might regulate transcription activity of PPARγ, and thus inhibiting the adipogenesis of FAPs. Therefore, the potential ESR1 binding sites on the PPARγ promoter were then predicted by JASPAR website (Figure 4C). To validate the biding site, we performed dual luciferase reporter assays by constructing transcription factor binding sites of the target gene promoter with predicted sequences of high scores and corresponding mutations in the pGL3‐basic vector (Figure 4D). Firefly luciferase was served as the reporter and Renilla luciferase as the internal control. Subsequently, FAPs were co‐transfected with WT or mutant pGL3‐PPARγ promoter reporter plasmids, along with the ESR1 overexpression plasmid, control Vector plasmid and pRL‐TK plasmid. Western blot analysis confirmed that ESR1 expression was effectively up‐regulated by the ESR1 overexpression plasmid in FAPs (Figure 4E,F). Overexpression of ESR1 (ESR1 OE) significantly inhibited the luciferase activity in PPARγ WT group when compared to PPARγ mutant (Mut) group (Figure 4G). Then the ChIP‐qPCR was applied to verify the capability of ESR1 to bind to PPARγ promoter. Significant increase in enrichment was detected in the samples incubated with anti‐ESR1 antibody when compared to those incubated with IgG, indicating that ESR1 could function as a transcription factor for PPARγ in FAPs (Figure 4H). Collectively, these findings suggested that ESR1 bound directly to the PPARγ promoter as a transcription factor, thereby inhibiting PPARγ transcription.

### 3.5 ESR1 enhanced expression of METTL3 in turn by serving as a transcription factor in FAPs

**FIGURE 5:**
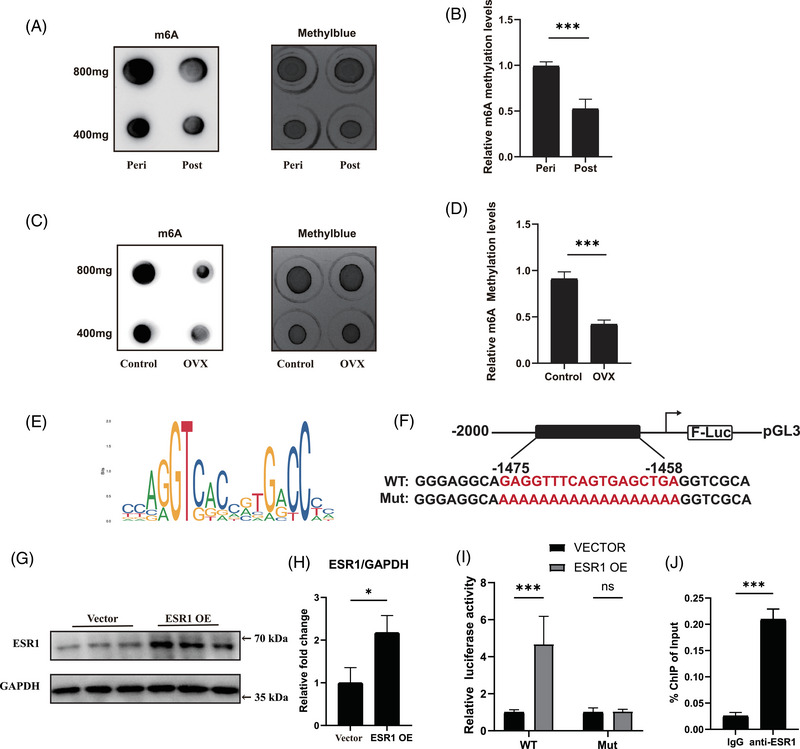
Oestrogen receptor 1 (ESR1) enhanced expression of methyltransferase‐like 3 (METTL3) in turn by serving as a transcription factor in fibro/adipogenic progenitors (FAPs). (A, B) Dot blot and quantitative assessment of relative N6‐methyladenosine (m6A) methylation levels in FAPs from peri‐menopausal (Peri) and post‐menopausal patients (Post; *n* = 3 patients/group). (C, D) Dot blot and quantitative assessment of relative m6A methylation levels in FAPs from sham mouse (Control) and FAPs from ovariectomy (OVX) mouse (*n* = 3 mice/group). (E) The sequence logo of potential ESR1 binding sites on the METTL3 promoter was predicted using JASPAR. (F) Scheme of wild type (WT) and mutant (Mut) pGL3‐METTL3 promoter reporter plasmids. (G, H) Protein levels and quantitative assessment of ESR1 and GAPDH in female FAPs transfected with vector (Vector) and ESR1 overexpression (ESR1 OE) plasmids (*n* = 3 per condition). (I) Relative luciferase activity after transfection of WT and mutant (Mut) pGL3‐METTL3 promoter reporter plasmids in female FAPs transfected with ESR1 overexpression plasmid (*n* = 3 per condition). (J) Chromatin immunoprecipitation (ChIP)‐qPCR for ESR1 binding to the METTL3 promoter in female FAPs under incubation with IgG or anti‐ESR1 antibodies (*n* = 3 per condition). Data were shown as mean ± standard deviation (SD), ns indicated no significant differences, * indicated *p* < .05, *** indicated *p* < .001.

Since oestrogen could down‐regulate expression of Mettl3 in kidney and liver tissues,^53^ we next investigated whether ESR1 acted as a transcription factor to regulate METTL3 expression in FAPs. We first tested the global m6A modification level of FAPs under E2 deficiency and found that the Overall m6A modification status decreased in female post‐menopausal and OVX‐FAPs, suggesting that decline in E2 reduced global m6A modification level (Figure 5A–D). Dual luciferase reporter experiment was then conducted to validate the binding of ESR1 to the METTL3 promoter. High‐scoring transcription factor binding sites and corresponding mutations were constructed in the pGL3‐basic vector based on prediction in JASPAR, with firefly luciferase as the reporter and Renilla luciferase as the internal control (Figure 5E,F). Next, WT or mutant pGL3‐METTL3 promoter reporter plasmids were co‐transfected with the ESR1 overexpression plasmid, control Vector plasmid and pRL‐TK plasmid into FAPs. Western blotting results demonstrated that the ESR1 overexpression plasmid effectively up‐regulated ESR1 expression in FAPs (Figure 5G,H). Overexpression of ESR1 (ESR1 OE) led to a significant increase in luciferase activity in the METTL3 WT group compared to the METTL3 mutant (Mut) group (Figure 5I). Subsequently, ChIP‐qPCR was conducted to validate the binding capability of ESR1 to the METTL3 promoter. A significant increase in enrichment was found in samples incubated with the anti‐ESR1 antibody compared to those incubated with the IgG antibody, suggesting that ESR1 could function as a transcription factor for METTL3 in FAPs (Figure 5J). Taken together, these findings suggested that ESR1 acted as transcription factor to enhanced the expression of METTL3.

### 3.6 E2 reactivated METTL3–ESR1‐mediated loop in female post‐menopausal FAPs

**FIGURE 6:**
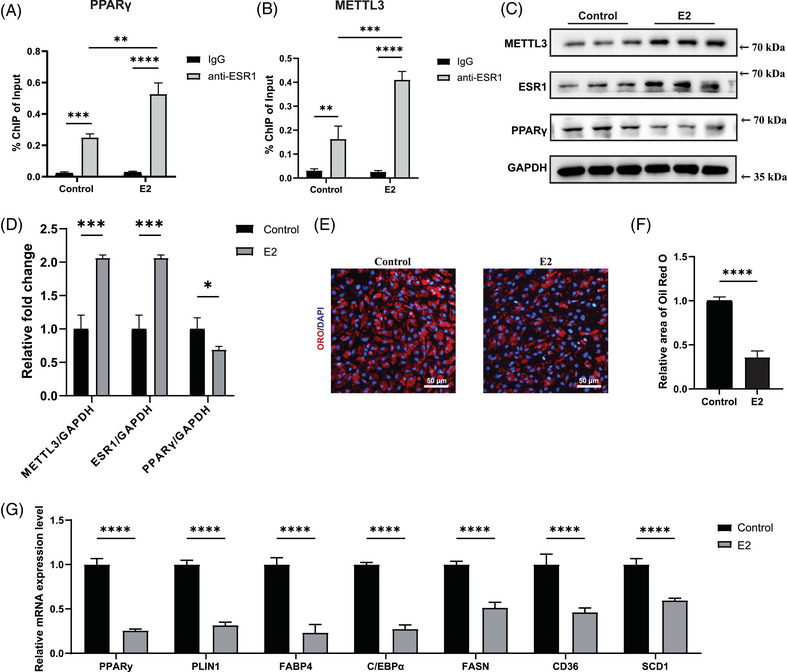
E2 reactivated methyltransferase‐like 3 (METTL3)–oestrogen receptor 1 (ESR1)‐mediated loop in female post‐menopausal fibro/adipogenic progenitors (FAPs). (A) Chromatin immunoprecipitation (ChIP)‐qPCR for ESR1 binding to the peroxisome proliferator‐activated receptor gamma (PPARγ) promoter in female FAPs incubated with IgG or anti‐ESR1 antibodies following 3‐day treatments with either DMSO (Control) or oestrogen (E2) (*n* = 3 per condition). (B) ChIP‐qPCR for ESR1 binding to the METTL3 promoter in female FAPs incubated with IgG or anti‐ESR1 antibodies following 3‐day treatments with either DMSO (Control) or oestrogen (E2) (*n* = 3 per condition). (C, D) The protein levels of ESR1 and METTL3 in female post‐menopausal FAPs treated with DMSO (Control) or oestrogen (E2) (*n* = 3 per condition). (E, F) Oil Red O staining and quantitative assessment of female post‐menopausal FAPs treated with DMSO (Control) or oestrogen (E2) following 10 days of adipogenic differentiation (*n* = 5 per condition). Red indicated Oil Red O, blue indicated DAPI and the merged images were shown. Scale bar, 50 µm. (g) The mRNA expression of adipogenic and lipogenic genes in female post‐menopausal FAPs treated with DMSO (Control) or oestrogen (E2) following 10 days of adipogenic differentiation (*n* = 3 per condition). Data were shown as mean ± standard deviation (SD), * indicated *p* < .05, ** indicated *p* < .01, *** indicated *p* < .001, **** indicated *p* < .0001.

Accumulating studies indicated that oestrogen regulated the expression of target genes via ESR1.^26^, ^27^, ^31^, ^54^ Thus, we then explored whether oestrogen could activate the METTL3–ESR1‐mediated loop in female post‐menopausal FAPs. We initially treated female post‐menopausal FAPs with E2 for 4 h. Following this treatment, ChIP‐qPCR was conducted to determine whether E2 treatment could enhance ESR1 binding to the PPARγ and METTL3 promoters. A significant increase in enrichment was observed in samples incubated with the anti‐ESR1 antibody after E2 treatment compared to the IgG group in both the PPARγ and METTL3 promoters, suggesting that E2 treatment effectively boosted ESR1's binding affinity to these promoter regions (Figure 6A,B). Subsequently, western blot revealed elevated expression levels of ESR1, METTL3 and PPARγ following E2 supplementation (Figure 6C,D). Oil Red O staining and RT‐qPCR were conducted after 10‐day induction in ADM with or without E2 treatment, and the results unveiled that E2 treatment suppressed adipogenic differentiation ability in post‐menopausal FAPs (Figure 6E–G). In summary, these data revealed that E2 could inhibit adipogenesis of female post‐menopausal FAPs by reactivating the METTL3–ESR1‐mediated loop.

### 3.7 E2 repletion reduced fatty infiltration and improved locomotor function in OVX mice through activating the METTL3–ESR1‐mediated loop

**FIGURE 7:**
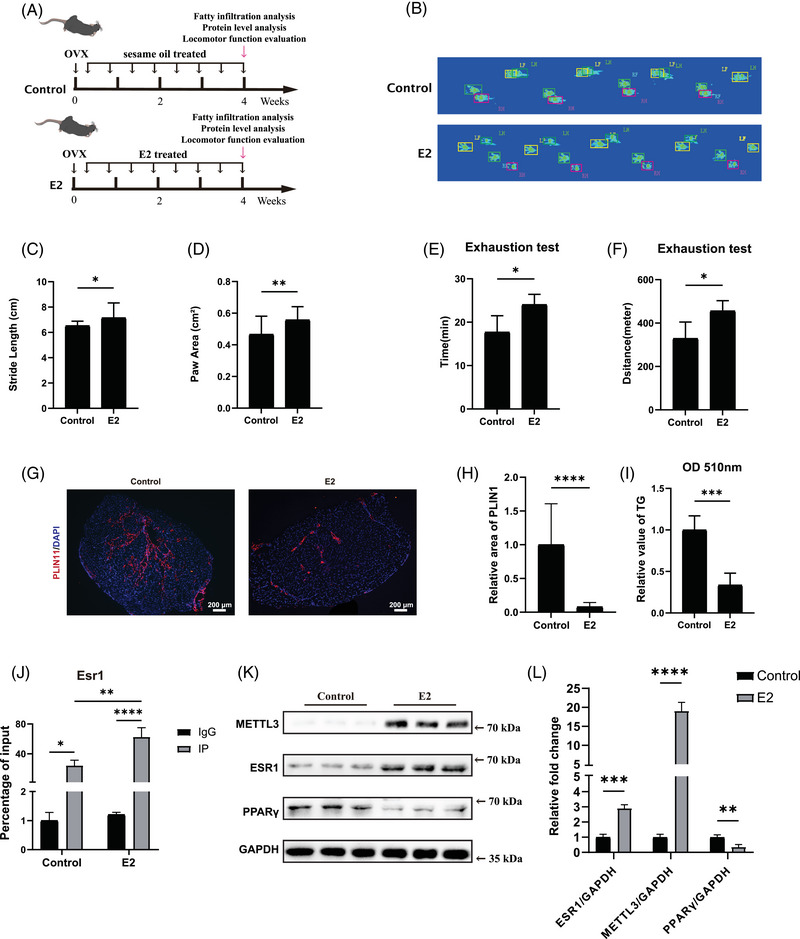
E2 treatment reduced fatty infiltration and improved locomotor function in ovariectomy (OVX) mice through activating the methyltransferase‐like 3 (METTL3)–oestrogen receptor 1 (ESR1)‐mediated loop. (A) Schematic diagram of the in vivo analysis of fatty infiltration, proteins level and locomotor function in OVX mice. OVX mice receiving daily subcutaneous injections of sesame oil were defined as the control group (Control), while OVX mice receiving sesame oil containing 17‐β‐estradiol‐3‐benzoate via subcutaneous injections were defined as the E2 group (E2). (B–D) Gait analysis including stride length and paw area of OVX mice from the control group and E2 group (*n* = 5 mice/group). (E, F) Treadmill tests of the control group (Control) and E2 group (E2) (*n* = 5 mice/group). (G, H) Immunofluorescence staining and quantitative assessment of fatty infiltration in the control group (Control) and E2 group (E2) (*n* = 5 mice/group). Red indicated PLIN1, blue indicated DAPI and the merged images were shown. Scale bar, 200 µm. (I) Quantification measurement of triglycerides in the supraspinatus muscles of the control group (Control) and E2 group (E2) (*n* = 5 mice/group). (J) MeRIP‐qPCR analysis of Esr1 between the anti‐IgG (IgG) group and anti‐N6‐methyladenosine (m6A) (IP) group in fibro/adipogenic progenitors (FAPs) of the control group (Control) and E2 group (E2) (*n* = 3 mice/group). (K, L) Protein levels and quantitative assessment of METTL3, ESR1, peroxisome proliferator‐activated receptor gamma (PPARγ) and GAPDH in the control group (Control) and E2 group (E2) (*n* = 3 mice/group). Data were shown as mean ± standard deviation (SD), * indicated *p* < .05, ** indicated *p* < .01, *** indicated *p* < .001, **** indicated *p* < .0001.

Muscular fatty infiltration significantly impaired motor and coordination functions.^5^, ^55^ Therefore, we then investigated whether E2 replenishment could mitigate fatty infiltration and improve locomotor function in vivo after menopause. OVX models were first established to simulate menopause at 8‐week‐old and then used for subsequent analyses at 12‐week‐old (Figure 7A). In the E2 treatment group, 150 µL of 18 µM 17‐β‐estradiol‐3‐benzoate was administered via daily subcutaneous injections. In the control group, 150 µL of sesame oil was administered daily without 17‐β‐estradiol‐3‐benzoate. Since gait analysis is a widely used method to assess locomotion ability,^41^, ^55^ it was performed to evaluate the locomotor function of involved models. In the gait analysis assay, stride length and paw contact area were recorded to reflect muscle strength and performance.^42^, ^43^ These parameters were significantly improved in OVX models with E2 treatment compared to control group (Figure 7B–D). Then the exhaustion test was conducted to assess locomotor performance. As expected, the OVX models in E2 treatment group also showed enhanced performance in running duration and distance relative to the control group (Figure 7E,F).

Subsequently, supraspinatus muscles from OVX mice were harvested for further assessments. Both PLIN1 staining and triglyceride quantification revealed less accumulated lipids in supraspinatus muscle after E2 treatment (Figure 7G–I). Then fresh primary FAPs were isolated to evaluate m6A modifications in Esr1 mRNA and protein level alterations of METTL3, ESR1 and PPARγ after E2 treatment. MeRIP‐qPCR was performed and the results suggested that E2 effectively increased m6A methylation of ESR1 segment targeting 717 bp site (Figure 7J). Western blot analysis demonstrated that E2 treatment significantly enhanced the expression of METTL3 and ESR1 while inhibiting the expression of PPARγ (Figure 7K,L), which further indicated activated Mettl3–Esr1‐mediated loop by E2 treatment. Taken together, these data demonstrated that E2 treatment contributed to mitigating muscular fatty infiltration and improving locomotor function in OVX model, which could be ascribed to activation of METTL3–ESR1‐mediated loop.

## 4 DISCUSSIONS

**FIGURE 8:**
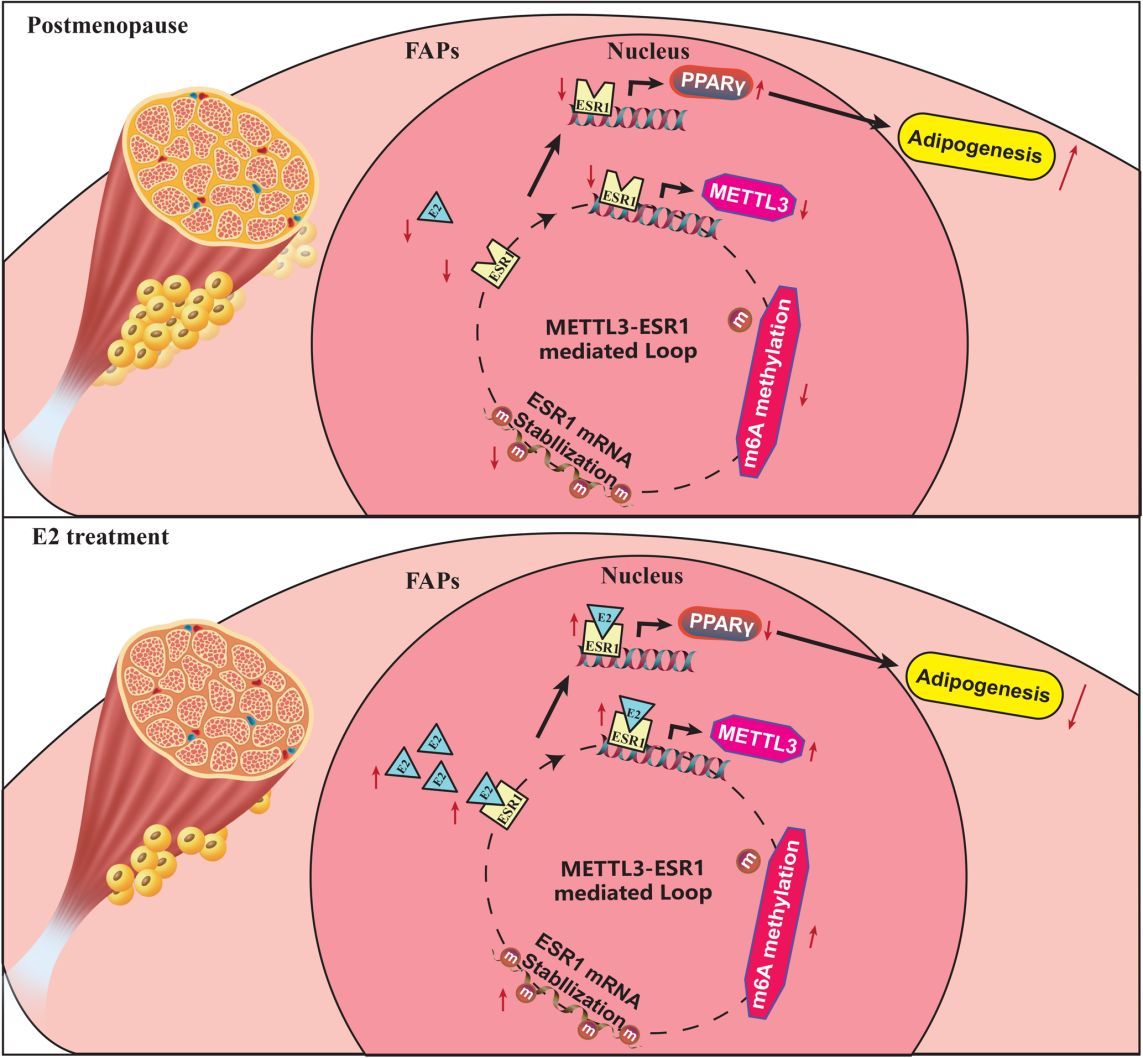
Schematic diagram of current study. Decreased expression of oestrogen receptor 1 (ESR1)/methyltransferase‐like 3 (METTL3) and increased adipogenesis ability in fibro/adipogenic progenitors (FAPs) was found in post‐menopausal female. METTL3‐mediated N6‐methyladenosine (m6A) methylation promoted ESR1 mRNA stability at the post‐transcriptional level in FAPs. METTL3‐mediated m6A modification promoted ESR1 expression by stabilising ESR1 mRNA, while ESR1 acted as a transcription factor that enhanced METTL3 transcription in turn. ESR1 also suppressed the transcription of the adipogenic transcription factor peroxisome proliferator‐activated receptor gamma (PPARγ), thereby inhibiting adipogenesis in FAPs. Reactivation of the METTL3–ESR1‐mediated loop by oestrogen alleviated excessive adipogenesis in FAPs from post‐menopausal women, and it also reduced muscular fatty infiltration, and improved locomotor function in ovariectomy (OVX) mice.

Our findings revealed that decreased expression of ESR1/METTL3 and increased adipogenesis ability in FAPs from post‐menopausal female. METTL3‐mediated m6A methylation promoted ESR1 mRNA stability at the post‐transcriptional level in FAPs. METTL3‐mediated m6A modification promoted ESR1 expression by stabilising ESR1 mRNA, while ESR1 acted as a transcription factor that enhanced METTL3 transcription in turn. ESR1 also suppressed the transcription of the adipogenic transcription factor PPARγ, thereby inhibiting adipogenesis in FAPs. Reactivation of the METTL3–ESR1‐mediated loop by oestrogen alleviated excessive adipogenesis in FAPs from post‐menopausal women, and it also reduced muscular fatty infiltration, and improved locomotor function in OVX mice (Figure 8).

Post‐menopausal muscular fatty infiltration significantly impaired mitochondrial function, increased muscular inflammation and exacerbated insulin resistance.^3^, ^29^, ^31^, ^56^ Accumulating evidences have shown that increased muscular fatty infiltration is associated with impaired locomotor function and poor prognosis in various diseases.^2^, ^3^, ^4^, ^56^ FAPs are the primary muscle‐resident cells for fatty infiltration in skeletal muscle.^1^, ^11^, ^12^, ^13^ Previous studies have shown that FAPs proliferated uncontrollably and tended towards adipogenesis in various muscular diseases, resulting in excessive muscular fatty infiltration.^1^, ^11^, ^12^, ^13^ Under normal physiological conditions, skeletal muscle injury initiates a temporary period of FAP proliferation, succeeded by apoptosis and subsequent phagocytic clearance, returning to baseline.^15^ However, in DMD, the lack of dystrophin leads to fragile muscle fibres, triggering a harmful cycle of muscle degeneration and repair.^12^ FAPs with pathological alterations fail to clear and return to quiescence, resulting in chronic inflammation, muscle fibrosis and fatty infiltration at the expense of muscle fibres.^12^ Therefore, numerous studies have attempted to alleviate fatty infiltration by regulating the proliferation and adipogenesis of FAPs.^9^, ^12^, ^13^, ^18^ Zhao et al. discovered that retinoic acid could inhibit FAPs adipogenesis via retinoic acid.^57^ Reggio et al. used the GSK3 inhibitor to suppress the WNT5a/GSK3/β‐catenin pathway, which further inhibited adipogenesis of FAPs.^58^ Furthermore, increasing evidence has suggested that interactions with immune cells play a vital role in the adipogenesis of FAPs.^12^, ^17^, ^59^, ^60^ Macrophages are critical regulators for FAP differentiation. Moratal et al. reported that IL‐4‐polarised macrophages enhanced the adipogenic potential of FAPs, whereas IL‐1β‐polarised macrophages effectively reduced fat formation in FAPs.^59^ IL‐4 has emerged as a crucial regulator of FAP fate. Recruited eosinophils could secrete IL‐4 to enhance IL‐4/IL‐13 signalling, preventing adipogenesis and promoting FAP proliferation within damaged muscle.^60^ Thus, these findings indicated that targeting FAPs could effectively treat excessive muscular fatty infiltration.

In the current study, we revealed decreased expression level of ESR1 in OVX‐FAPs and female post‐menopausal FAPs, contributing to enhanced adipogenic differentiation potential. ESR1 not only functions as a nuclear receptor but also acts as a transcription factor, activated by oestrogen and binding to target gene promoters to regulate transcription.^26^, ^29^, ^30^ Research have demonstrated that oestrogen played an essential role in maintaining muscle homeostasis, balancing metabolism^26^, ^27^, ^28^, ^30^, ^31^, ^61^ and inhibiting adipogenesis in various cells.^26^, ^48^, ^49^, ^50^, ^51^ Oestrogen achieves its function by binding to ESR in the vast majority of cases. An remarked decease of ESR1 has also been observed in post‐menopause, which significantly contributed to muscle atrophy, sarcopenia and lipid abnormality.^27^, ^31^ However, there is limited knowledge about how ESR1 affects excessive fatty infiltration in female post‐menopausal muscle. The current study revealed that ESR1 could inhibit the transcription of the key adipogenic gene PPARγ, acting as a transcription factor and thereby suppressing adipogenesis of FAPs. These finding further added the knowledge of how ESR1 played its role in excessive muscular fatty infiltration in post‐menopausal women.

m6A modification stands as one of the most widespread and influential mRNA epigenetic alterations, significantly impacting mRNA transcription, stability and translation.^62^, ^63^, ^64^ METTL3 functions as part of the m6A methyltransferase complex, which plays a pivotal role in cell differentiation, development and stress response.^62^, ^65^, ^66^ Mettl3‐mediated m6A modifications in eukaryotic RNA plays a critical role in regulating cell fate determination of stem cell.^67^, ^68^, ^69^ Wu et al. suggested that the loss of Mettl3 in bone marrow mesenchymal stem cells disrupted cell fate, leading to impaired osteogenic potential and enhanced adipogenic potential.^65^ Giordani et al. revealed that epigenetic alternations affected FAPs behaviour during the progression of fatty infiltration.^69^ Therefore, epigenetic changes might regulate muscular fatty infiltration in post‐menopause through a specific mechanism. In this study, we found that post‐menopausal female FAPs exhibited significant epigenetic modifications along with decreased expression of ESR1. As far as we are aware, this was the first research to demonstrate that METTL3‐mediated m6A modification enhanced ESR1 expression, thereby inhibiting adipogenesis in FAPs. The decline in the interplay between METTL3 and ESR1 exacerbated excessive muscular fatty infiltration in post‐menopausal women. Thus, epigenetic regulation played a critical role in post‐menopausal fatty infiltration, and the METTL3–ESR1‐mediated loop may offer a potential therapeutic target.

Hormone replacement therapy is frequently used to treat menopausal symptoms.^26^, ^30^ Previous studies have demonstrated that oestrogen repletion therapy benefits energy homeostasis, improves fat distribution and reduces insulin resistance and inflammation in muscle.^26^, ^30^ However, its role in post‐menopausal muscular fat infiltration remains unexplored. Given that oestrogen deficiency is a major physiological change during menopause,^21^ our study confirmed the involvement of ESR1 as a key gene in post‐menopausal muscle fatty infiltration. Addition of oestrogen could reactive METTL3–ESR1‐mediated loop, inhibit excessive adipogenesis of FAPs and thus mitigating muscular fatty infiltration and improving locomotor function in OVX mice. Therefore, oestrogen supplementation could be a promising treatment for this condition. The current study provided preliminary in vivo data from OVX mouse on the efficacy of oestrogen replacement therapy in treating fat infiltration post‐menopause, thereby expanding its clinical prospects. Clinical studies are further needed to establish the utilisation, safety and feasibility of oestrogen repletion in inhibiting post‐menopausal muscular fatty infiltration.

There are several limitations in our study. First, while some studies have demonstrated that aging increased the prevalence and severity of muscular fatty infiltration, others have not found such an association.^70^ Differences in age range, assessment methods and physiological changes may contribute to these conflicting results.^70^ To address these potential confounders, we used an OVX model and recruited peri‐menopausal and post‐menopausal women with no significant age differences to specifically focus on the role of estradiol in muscular fatty infiltration. We believe that muscle fatty infiltration worsens with aging, and further studies are needed to investigate how aging affects muscular fatty infiltration. Additionally, numerous studies have reported gender differences in muscle fatty infiltration.^9^, ^71^, ^72^, ^73^ Healthy elderly women exhibit more severe muscular fatty infiltration than men.^73^ Female patients with rotator cuff injuries experience greater severity of muscular fatty infiltration.^9^, ^71^, ^72^ This phenomenon aggravated with decreased oestrogen levels after menopause,^74^ highlighting the critical role of oestrogen in inhibiting muscular fatty infiltration. Furthermore, gender differences existed in muscle characteristics, including the ratio of fibre types I and II, variations in capillary supply and differences in insulin sensitivity. Therefore, it would be interesting to further explore gender differences in muscular fatty infiltration and how sex hormones influence this process.

## AUTHOR CONTRIBUTIONS

Hao Zhou, Xingzuan Lin and Xiexiang Shao designed the study, collected the experimental data and wrote the original draft. Siyuan Zhu and Xiexiang Shao conducted the image analysis, statistical analysis and wrote the original draft. Siyuan Zhu, Han Zhou and Shujing Feng designed the study, reviewed and edited the manuscript. Xiexiang Shao and Hao Zhou reviewed and edited the manuscript. Jianhua Wang and Xiexiang Shao administrated the project and edited the manuscript. Ru Wang, Jinkui Cai and Yongmin Cao conducted MeRIP‐seq and subsequent analysis. Jianhua Wang, Xiexiang Shao and Ru Wang should be considered as co‐corresponding author. Hao Zhou, Shujing Feng, Jinkui Cai and Xiexiang Shao contributed equally and should be considered as co‐first author.

## CONFLICT OF INTEREST STATEMENT

The authors declare no conflicts of interest.

## ETHICS STATEMENT

The study was approved by local ethical Committee (Approval No. XHEC‐F‐2023‐029, Approval No. XHEC‐D‐2022‐129 and Approval No. XHEC‐D‐2024‐073‐1)

## CONSENT FOR PUBLICATION

All authors agreed to publish the current study in *Cellular & Molecular Biology Letters*.

## Supporting information



Supporting Information

## Data Availability

Data supporting the findings of this study are available from the corresponding author upon request.
