# Supplementary material for: Oestrogen suppresses the adipogenesis of fibro/adipogenic progenitors through reactivating the METTL3–ESR1‐mediated loop in post‐menopausal females
Source: Clin Transl Med. 2025 Jan 28;15(2):e70206. doi: 10.1002/ctm2.70206 (PMC11774659; doi:10.1002/ctm2.70206)
Supplement: Supplementary file 1 — Supporting Information [file CTM2-15-e70206-s001.docx]

**Supplemental files**

**Supplemental Table 1.** **Subject characteristics of women participants**

|  | **Perimenopausal Female** | **Postmenopausal Female** |  |
| --- | --- | --- | --- |
| **Phenotype** |  |  | ***P*value** |
| **Serum estradiol (nM)** | 0.303±0.038 | 0.156±0.014 | <0.0001 |
| **Age (yr)** | 50.9±1.5 | 52.9±1.8 | 0.0517 |
| **BMI (kg/m²)** | 23.3±1.9 | 24.4±1.6 | 0.4804 |
| **Glucose (mmol/l)** | 5.5±1.4 | 6.1±0.7 | 0.8602 |

| **Supplemental Table 2. Primer sequences for RT-qPCR** | |
| --- | --- |
| **Target gene** | **Sequences** |
| Human GAPDH-F | 5′-CAAGGCTGAGAACGGGAAGC-3 |
| Human GAPDH-R | 5′-AGGGGGCAGAGATGATGACC-3′ |
| Human PPARγ-F | 5′-CCAGAAGCCTGCATTTCTGC-3′ |
| Human PPARγ-R | 5′-CACGGAGCTGATCCCAAAGT-3′ |
| Human PLIN1-F | 5'-TGTGCAATGCCTATGAGAAGG-3' |
| Human PLIN1-R | 5'-AGGGCGGGGATCTTTTCCT-3' |
| Human C/EBPα-F | 5'-TATAGGCTGGGCTTCCCCTT-3' |
| Human C/EBPα-R | 5'-AGCTTTCTGGTGTGACTCGG-3' |
| Human FABP4-F | 5'-ACTGGGCCAGGAATTTGACG-3' |
| Human FABP4-R | 5'-CTCGTGGAAGTGACGCCTT-3' |
| Human ESR1-F | 5'-GAAAGGTGGGATACGAAAAGACC-3' |
| Human ESR1-R | 5'-GCTGTTCTTCTTAGAGCGTTTGA-3' |
| Human METTL3-F | 5'-TTGTCTCCAACCTTCCGTAGT-3' |
| Human METTL3-R | 5'-CCAGATCAGAGAGGTGGTGTAG-3' |
| Human CD36-F | 5'-TTGATTGAAAAATCCTTCTTAGCCA-3' |
| Human CD36-R | 5'-TGGTTTCTACAAGCTCTGGTTCTTA-3' |
| Human FASN-F | 5'-AAGGACCTGTCT AGGTTTGATGC-3' |
| Human FASN-R | 5'-TGGCTTCATAGGTGACT TCCA-3' |
| Human SCD1-F | 5'-TTCCCGACGTGGCTTTTTCT-3' |
| Human SCD1-R | 5'-AGCCAGGTTTGTAGTACCTCC-3' |
| Mouse Gapdh-F | 5′-AGGTCGGTGTGAACGGATTTG-3 |
| Mouse Gapdh-R | 5′-GGGGTCGTTGATGGCAACA-3′ |
| Mouse Pparγ-F | 5′-GGAAGACCACTCGCATTCCTT-3′ |
| Mouse Pparγ-R | 5′-GTAATCAGCAACCATTGGGTCA-3′ |
| Mouse Plin1-F | 5'-CTGTGTGCAATGCCTATGAGA-3' |
| Mouse Plin1-R | 5'-CTGGAGGGTATTGAAGAGCCG-3' |
| Mouse C/ebpα-F | 5'-GCGGGAACGCAACAACATC-3' |
| Mouse C/ebpα-R | 5'-GTCACTGGTCAACTCCAGCAC-3' |
| Mouse Fabp4-F | 5'-AAGGTGAAGAGCATCATAACCCT-3' |
| Mouse Fabp4-R | 5'-TCACGCCTTTCATAACACATTCC-3' |
| Mouse Esr1-F | 5'-CCTCCCGCCTTCTACAGGT-3' |
| Mouse Esr1-R | 5'-CACACGGCACAGTAGCGAG-3' |
| Mouse Mettl3-F | 5'-CTGGGCACTTGGATTTAAGGAA-3' |
| Mouse Mettl3-R | 5'-TGAGAGGTGGTGTAGCAACTT-3' |
| Mouse Mettl14-F | 5'-GAGCTGAGAGTGCGGATAGC-3' |
| Mouse Mettl14-R | 5'-GCAGATGTATCATAGGAAGCCC-3' |
| Mouse Mettl16-F | 5'-CAAGGACAAACCACCTGACTT-3' |
| Mouse Mettl16-R | 5'-GTGGGAATTAGTCTCTCCAAAGG-3' |
| Mouse Wtap-F | 5'-ATGGCACGGGATGAGTTAATTC-3' |
| Mouse Wtap-R | 5'-TTCCCTTAAACCAGTCACATCG-3' |
| Mouse Kiaa1429-F | 5'-GGTTCGTTTTCCGTGTGTGG-3' |
| Mouse Kiaa1429-R | 5'-GCCACTATGGGCTCGTACTC-3' |
| Mouse Cd36-F | 5'-TGTGGAGCAACTGGTGGATG-3' |
| Mouse Cd36-R | 5'-CGTGGCCCGGTTCTAATTCA-3' |
| Mouse Fasn-F | 5'-GCAGCTGTTGGTTTGTCCTG-3' |
| Mouse Fasn-R | 5'-ATTCACTGCAGCCTGAGGTC-3' |
| Mouse Scd1-F | 5'-GTTCCGCCACTCGCCTAC-3' |
| Mouse Scd1-R | 5'-TGTAAGAACTGGAGATCTCTTGGA-3' |

| **Supplemental Table 3. Primer sequences for MeRIP-qPCR** | |
| --- | --- |
| **Target gene** | **Sequences** |
| Mouse Esr1-seg1-F | 5′-TTCTGACAATCGACGCCAGAA-3′ |
| Mouse Esr1-seg1-R | 5′-TCTTAAAGAAAGCCTTGCAGCC-3′ |
| Mouse Esr1-seg2-F | 5′-GATAAGCACTTCATAATGGCTCCA-3′ |
| Mouse Esr1-seg2-R | 5′-CATGTTGCTATAGGAATGCAAGC-3′ |
| Mouse Esr1-seg3-F | 5′-GTCACAATGAACCTGCAAGC-3′ |
| Mouse Esr1-seg3-R | 5′-ATTCTCCACATTTCTCCCTTACT-3′ |
| Mouse Esr1-seg4-F | 5′-GAGTCCTTTGAACAAGGGGAT-3′ |
| Mouse Esr1-seg4-R | 5′-CCCATCATATCTCAATGGAGTTC-3′ |
| Mouse Esr1-seg5-F | 5′-TAGCTAATGGGTCAGTGGGTTCT-3′ |
| Mouse Esr1-seg5-R | 5′-AGATGGGATAATGTAAAACCCTCC-3′ |

| **Supplemental Table 4. Primer sequences for ChIP-qPCR** | |
| --- | --- |
| **Target gene** | **Sequences** |
| Human PPAR-F | 5′-TAGCTAATGGGTCAGTGGGTTCT-3′ |
| Human PPAR-R | 5′-GAGCCACGGATCTTACAGGG-3′ |
| Human METTL3-F | 5′-TAGCTAATGGGTCAGTGGGTTCT-3′ |
| Human METTL3-R | 5′-GAGCCACGGATCTTACAGGG-3′ |


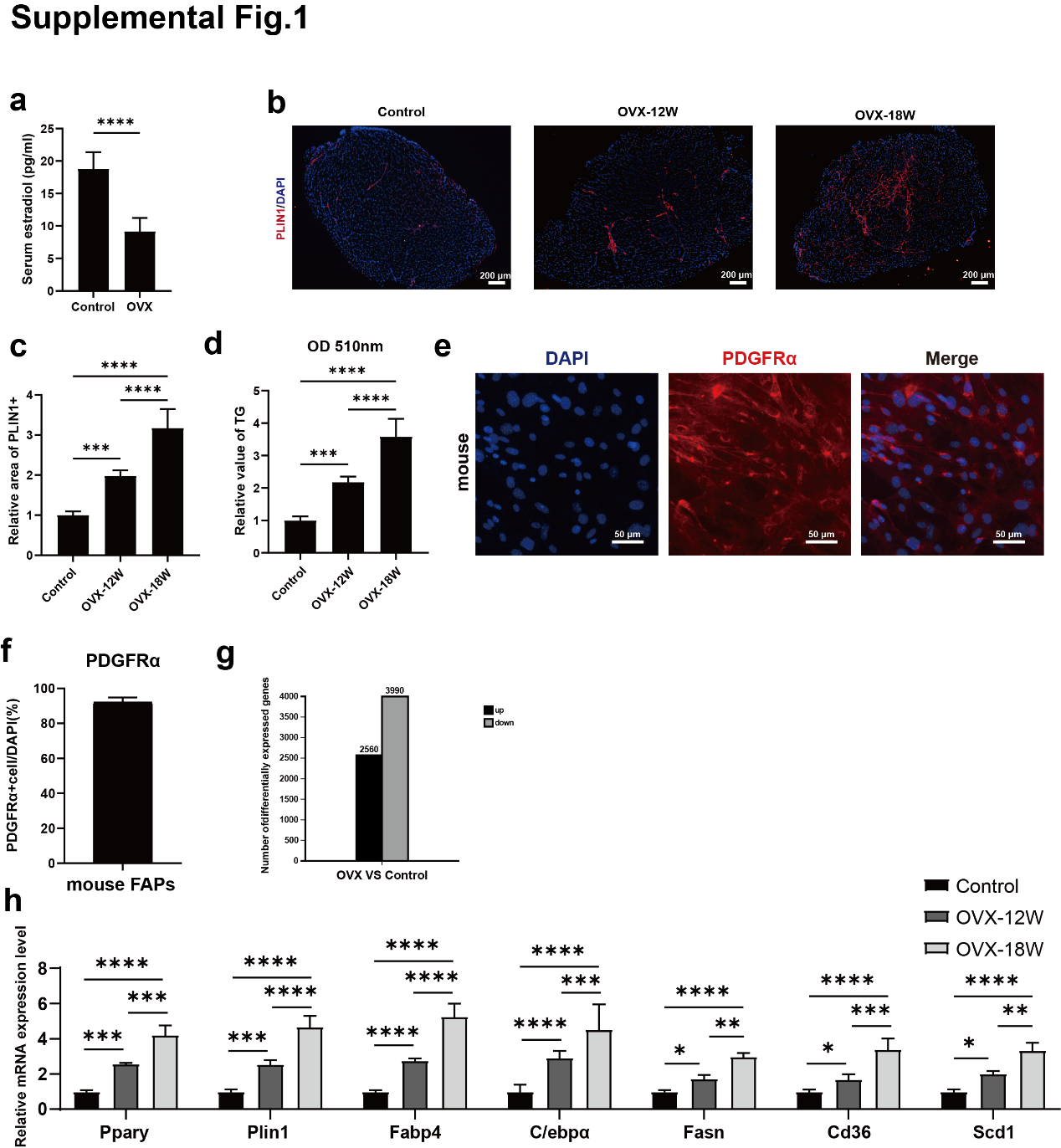


**Supplemental Fig.1**

**Excessive muscular fatty infiltration was confirmed in ovariectomy mouse.**

(a) Estradiol level in serum from sham mice (Control) and ovariectomy mice (OVX) (n = 8 mice/group).

(b-c) Immunofluorescence staining of PLIN1 and quantification analysis of lipid droplets in the supraspinatus muscles of sham mice (Control), OVX mice euthanized at 12 weeks (OVX-12W), and at 18 weeks (OVX-18W) (n = 4 mice/group). Red indicated PLIN1, blue indicated DAPI, and the merged images were shown. Scale bar, 200 μm.

(d) Quantification measurement of triglycerides in the supraspinatus muscle of sham mice (Control), OVX mice euthanized at 12 weeks (OVX-12W), and at 18 weeks (OVX-18W) (n = 5 mice/group).

(e-f) Immunofluorescence staining and quantification analysis of mouse FAPs (n = 5 mice/group). Red indicated PDGFRα, blue indicated DAPI, and the merged images were shown. Scale bar, 50 μm.

(g) Bar chart of differentially expressed genes in FAPs from ovariectomy mouse (OVX) and sham mouse (Control).

(h) Relative mRNA expression of adipogenic and lipogenic genes in FAPs from sham mouse (Control) and FAPs from ovariectomy mouse (OVX). (n = 3 mice/group).

Data were shown as mean ± SD, *indicated P<0.05, ** indicated P < 0.01, *** indicated P < 0.001, **** indicated P < 0.0001.


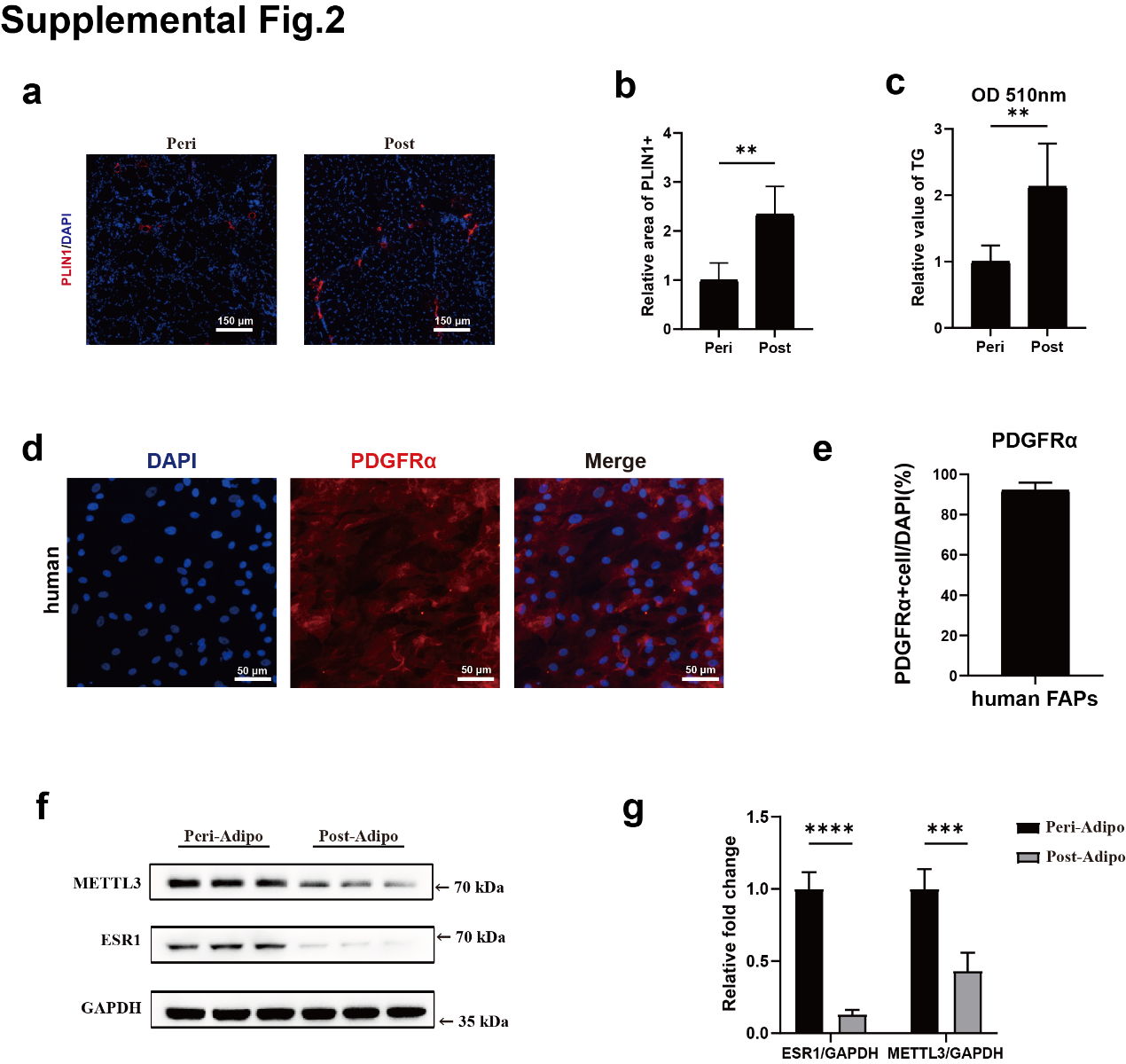


**Supplemental Fig. 2**

**Excessive muscular fatty infiltration was confirmed in female postmenopausal women.**

(a-b) Immunofluorescence staining of PLIN1 and quantification analysis of lipid droplets in the supraspinatus muscles of perimenopausal women (Peri) and postmenopausal women (Post) (n = 4 patients/group). Red indicated PLIN1, blue indicated DAPI, and the merged images were shown. Scale bar, 150 μm.

(c) Quantification measurement of triglycerides in the supraspinatus muscle of perimenopausal women (Peri) and postmenopausal women (Post) (n = 5 patients/group).

(d-e) Immunofluorescence staining and quantification analysis of human FAPs (n = 5 patients/group). Red indicated PDGFRα, blue indicated DAPI, and the merged images were shown. Scale bar, 50 μm.

(f-g) Protein levels and quantitative assessment of ESR1, METTL3, and GAPDH after adipogenic differentiation of FAPs from perimenopausal (Peri-Adipo) and postmenopausal women (Post-Adipo) (n = 3 patients/group).

Data were shown as mean ± SD. ** indicated P < 0.01, *** indicated P < 0.001, **** indicated P < 0.0001.


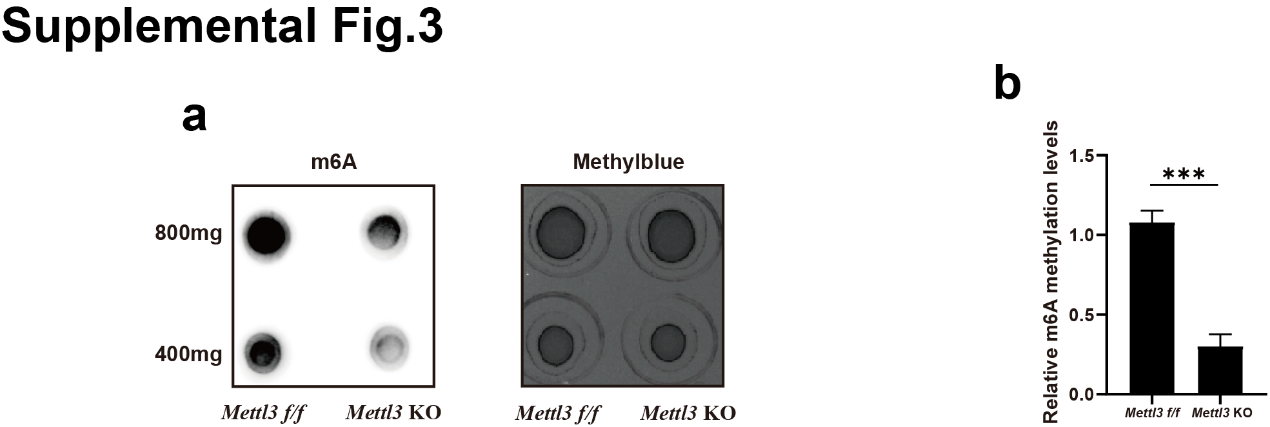


**Supplemental Fig. 3**

**Global m6A methylation levels after FAPs specific *Mettl3* knockout in FAPs**

(a-b) Dot blot and quantification analysis of relative global m6A methylation levels in *Mettl3* knockout (KO) FAPs and *Mettl3 flox/flox (f/f)* FAPs (n = 3 mice/group).

Data were shown as mean ± SD, *** indicated P < 0.001.


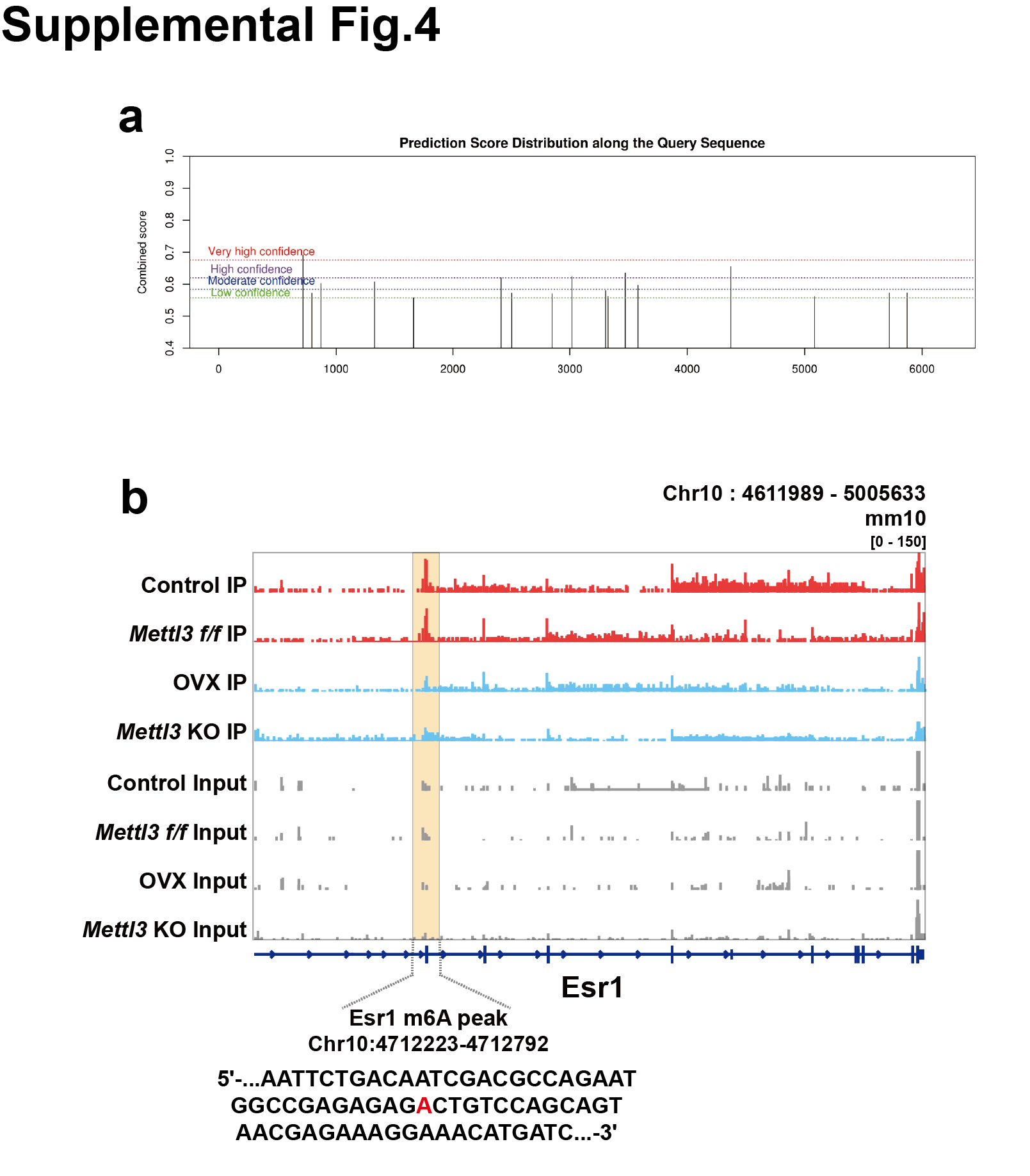


**Supplemental Fig. 4**

**Potential m6A methylation sites in ESR1 mRNA**

1. Potential m6A methylation sites in ESR1 mRNA predicted by SRAMP.

(b) Visualization of the m6A peaks in Esr1 gene by MeRIP-seq in Control, OVX, *Mettl3 f/f*, and *Mettl3* KO FAPs.


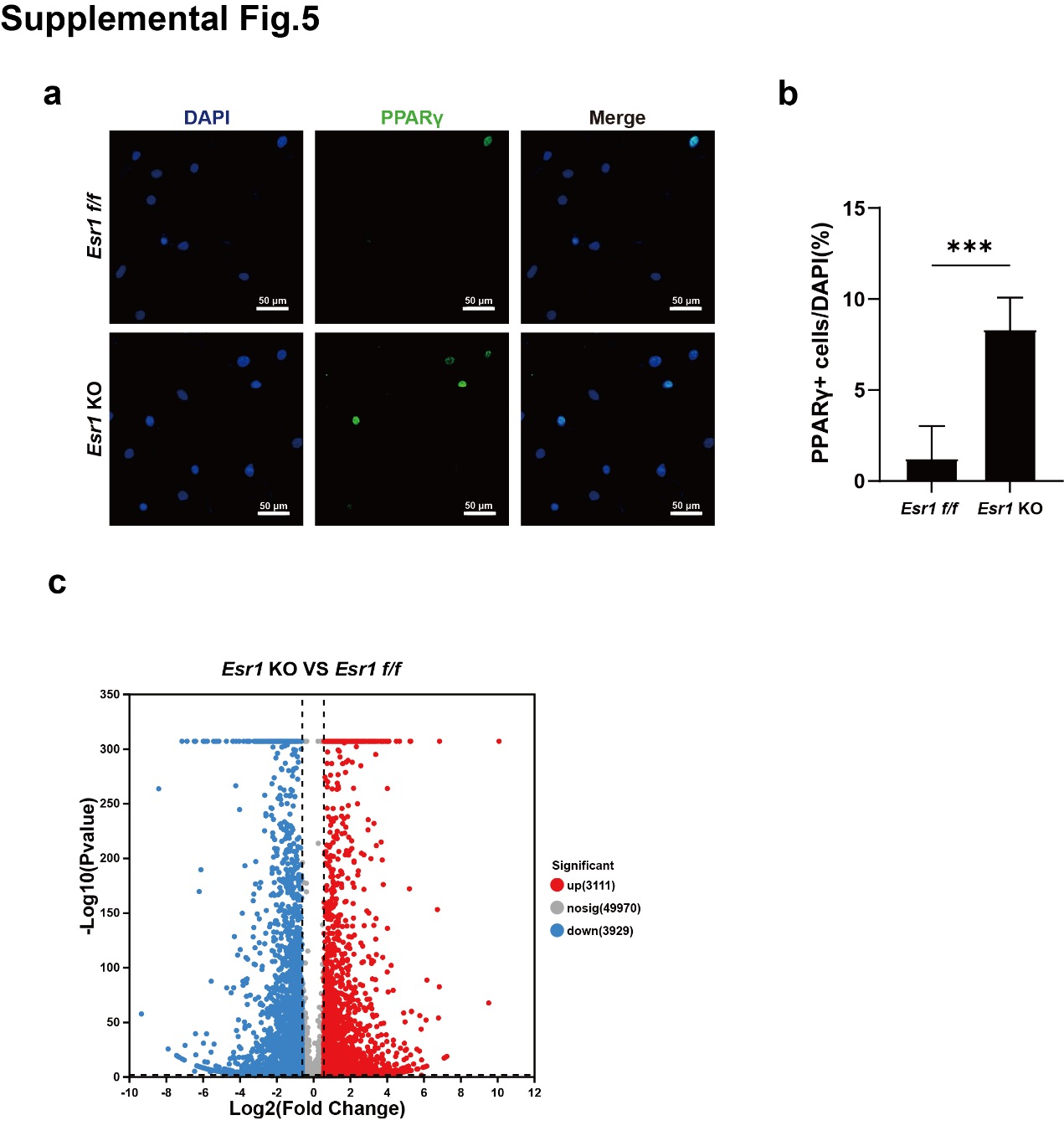


**Supplemental Fig. 5**

**ESR1 suppressed adipogenic differentiation of FAPs by inhibiting transcription of PPARγ**

(a-b) Immunofluorescence staining of PPARγ and quantitative assessment of FAPs from *Esr1 f/f* mice and *Esr1* KO mice (n = 4 mice/group). Green indicated PPARγ, blue indicated DAPI, and the merged images were shown. Scale bar, 50 μm.

(c) Volcano plot of differentially expressed genes in *Esr1* KO FAPs and *Esr1 f/f* FAPs.

Data were shown as mean ± SD, *** indicated P < 0.001.
